# Diverse set of microRNAs are responsive to powdery mildew infection and heat stress in wheat (*Triticum aestivum *L.)

**DOI:** 10.1186/1471-2229-10-123

**Published:** 2010-06-24

**Authors:** Mingming Xin, Yu Wang, Yingyin Yao, Chaojie Xie, Huiru Peng, Zhongfu Ni, Qixin Sun

**Affiliations:** 1 State Key Laboratory for Agrobiotechnology and Key Laboratory of Crop Heterosis and Utilization (MOE) and Key Laboratory of Crop Genomics and Genetic Improvement (MOA), Beijing Key Laboratory of Crop Genetic Improvement, China Agricultural University, Beijing, 100094, China; 2 National Plant Gene Research Centre (Beijing), Beijing 100094, China; 3 Department of Plant Genetics & Breeding, China Agricultural University, Yuanmingyuan Xi Road No. 2, Haidian District, Beijing, 100193, China

## Abstract

**Background:**

MicroRNAs (miRNAs) are a class of small non-coding regulatory RNAs that regulate gene expression by guiding target mRNA cleavage or translational inhibition. MiRNAs can have large-scale regulatory effects on development and stress response in plants.

**Results:**

To test whether miRNAs play roles in regulating response to powdery mildew infection and heat stress in wheat, by using Solexa high-throughput sequencing we cloned the small RNA from wheat leaves infected by preponderant physiological strain *Erysiphe graminis f. sp. tritici *(*Egt*) or by heat stress treatment. A total of 153 miRNAs were identified, which belong to 51 known and 81 novel miRNA families. We found that 24 and 12 miRNAs were responsive to powdery mildew infection and heat stress, respectively. We further predicted that 149 target genes were potentially regulated by the novel wheat miRNA.

**Conclusions:**

Our results indicated that diverse set of wheat miRNAs were responsive to powdery mildew infection and heat stress and could function in wheat responses to both biotic and abiotic stresses.

## Background

Wheat (*Triticum aestivum*, AABBDD, 2n = 42) is the most widely grown crop plant, occupying 17% of all the cultivated land, provide approximately 55% of carbohydrates for world human consumption [1], Biotic and abiotic stresses are important limiting factors for yield and grain quality in wheat production. For instance, powdery mildew, caused by the obligate biotrophic fungus *Blumeria graminis f. sp. tritici *(*Bgt*), is one of the most devastating diseases of wheat in China and worldwide, and causing significant yield losses [2,3]. High temperature, often combined with drought stress, causes yield loss and reduces the grain quality [4]. To reduce the damage caused by biotic and abiotic stresses, plants have evolved sophisticated adaptive response mechanisms to reprogram gene expression at the transcriptional, post-transcriptional and post-translational levels [5]. Therefore, transcript profiling have been successfully employed to determine the transcriptional responses to powdery mildew infection and heat stress in wheat, and the results revealed that a number of genes were significantly induced or repressed in response to these stresses [6,7]. In addition, much progress has been made in unraveling the complex post-transcriptional regulation mechanisms in response to stress. Recently discovered microRNAs (miRNAs) and endogenous small interfering RNAs (siRNAs) have emerged as important posttranscriptional regulators in plant stress responses [8]. However, no report has been published on the roles of small RNAs in wheat response to abiotic and biotic stresses.

MicroRNAs (miRNAs) are a class of small RNAs that serve as posttranscriptional negative regulators of gene expression in plants and animals [9-14]. Up to date, plant miRNAs have been shown to function in many plant processes, including developmental transitions [15,16], leaf growth [17], organ polarity[18], auxin signaling [19] and RNA metabolism [20-22]. Importantly, increasing evidence indicated that miRNAs also play important roles in plant response to abiotic and biotic stresses [8]. The observation that some of the miRNAs are up- or down- regulated in response to stress implies that these miRNAs could play important roles in stress tolerance [5]. For example, in *Arabidopsis*, miR393 and other miRNAs are induced by cold stress [22,23]. In rice, miR169g and miR393 are up-regulated under drought stress [24]. *Arabidopsis *miR398 directs the cleavage of CSD1 and CSD2 mRNA under normal conditions, and down-regulation of miR398 by oxidative stress results in accumulation of CDS1 and CSD2 mRNAs [25]. Recent studies in *Arabidopsis *have also established that miR399, miR395 and miR398 are induced in response to phosphate-, sulfate- and Cu^2+^-deprived conditions, respectively [8,26-29]. In addition, among the 42 *Populus *miRNA families, expression of some miRNAs are altered in response to cold, heat, salinity, dehydration, and mechanical stresses [30]. Pathogenic bacteria, fungi, viruses, insect pests and nematodes cause severe damage to plants [5]. Recent discovery also revealed that miR393-guided post-transcriptional regulation plays a crucial role in the plant defense against pathogens through targeting transport inhibitor response 1 (TIR1), an auxin receptor [31]. Transgenic *Arabidopsis *overexpressing miR393a shows enhanced resistance to virulent *P. syringae *pv. tomato [5]. Another report in loblolly pine indicated that expressions of 10 miRNAs are decreased in response to the rust fungus [32]. Functional analyses have demonstrated that several plant miRNAs play vital roles in plant resistance to abiotic as well as biotic stresses [4,33-37].

Although some of the stress-responsive miRNA families are deeply conserved among various plant species, including *Arabidopsis*, rice and *Populus*, their species-specific function may be the results of adaptation to long-term growth and survival in stressful environment [30]. Moreover, some species-specific miRNAs could also play possible roles in the regulatory networks associated with the stress resistance, and individual miRNAs of a family response differentially to stress, suggesting that different members in same miRNA family may have different functions [30]. Recently, a total of 58 miRNAs comprising 43 miRNA families have been cloned in wheat [38], however, their expression level in response to abiotic and biotic stress are still unknown. With the development of high-throughput sequencing technology, it became possible to discover several species-specific or lowly expressed miRNAs and different members in the same miRNA family. In this study, by using Solexa high-throughput sequencing, we indentified a diverse set of wheat small RNAs which are responsive to powdery mildew infection and heat stress. A total of 51 known conserved miRNAs and 81 new identified miRNAs were obtained, increasing the number of wheat miRNA families from 43 to 132. Moreover we also found that many of these wheat miRNAs showed differential expression in response to powdery mildew infection and heat stress. In addition, 149 genes were predicted as potential targets for novel wheat miRNAs, which included transcription factors implicated in development as well as genes involved in other physiological processes, such as stress responses.

## Results

### High-throughput sequencing of wheat small RNAs

To investigate the role of wheat small RNA in powdery mildew resistance and heat stress, six small RNA libraries were constructed and sequenced. Disease susceptible wheat cultivar Jingdong8 (JD8) and its near-isogenic resistant line Jingdong8-*Pm30 *(JD8-*Pm30*) with single resistant gene *Pm30 *were used for powdery mildew infection and the preponderant physiological strain *Erysiphe graminis f. sp. tritici *(*Egt*) (Isolate E09) was used for powdery mildew infection. Non-inoculated and inoculated seedlings of JD8 and JD8-*Pm30 *were used to construct four small RNA libraries, designated JD8, JD8-*Egt*, JD8-*Pm30 *and JD8-*Pm30*-*Egt*, respectively. As for heat stress treatment, seedlings of heat tolerant cultivar TAM107 that undergone 40°C for 1 hour and those grown in normal condition were selected to build small RNA libraries, designated TAM107 and TAM107-heat, respectively.

These six wheat small RNA libraries were sequenced by Solexa high-throughput sequencing, and the numbers of raw reads for JD8, JD8-*Egt*, JD8-*Pm30*, JD8-*Pm30*-*Egt*, TAM107 and TAM107-heat were 3101553, 3848063, 2664384, 2560124, 4036437 and 4802099, respectively. To simplify the sequencing data, the identical sequence reads among the 6 small RNA libraries were grouped and converted into unique sequences. After removing the low quality reads, a total of 3839724 unique sequences ranging from 18-30 nt in length were obtained. Theoretically, these sequences could cover most of the small RNAs in our six samples used in this study.

**Figure 1:**
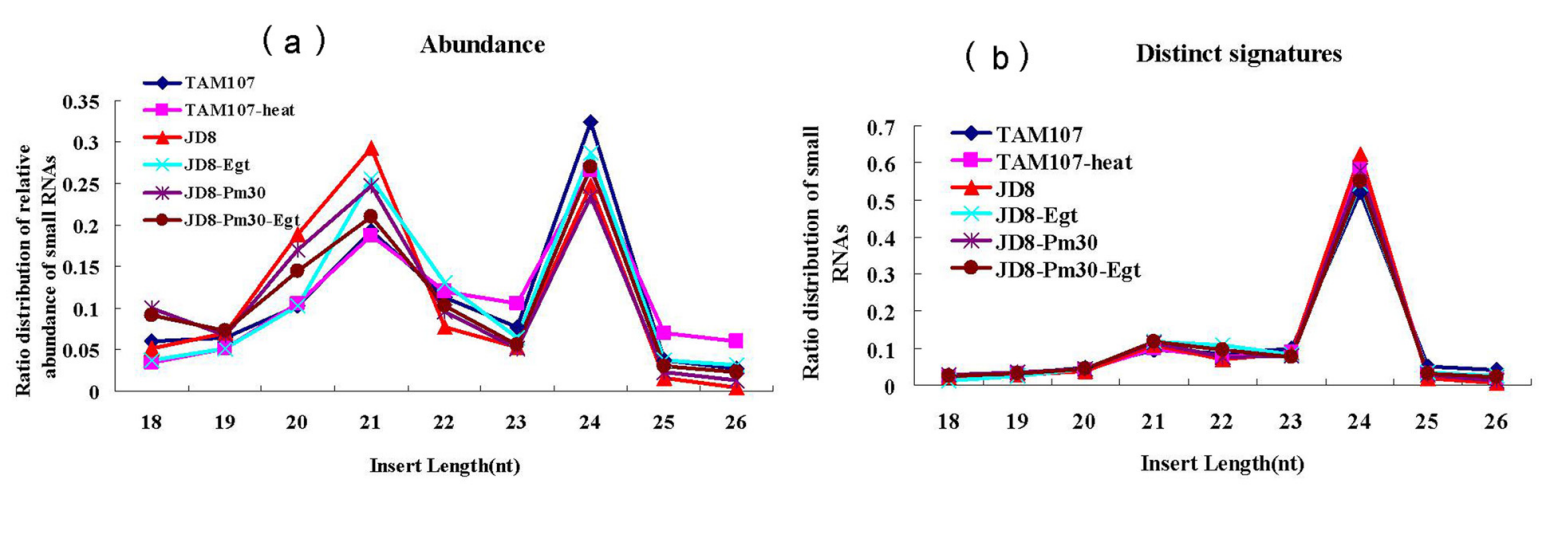
**small RNA size distribution with Solexa's high-throughput sequencing**. a) Total abundance of sequences vs. size. b) Number of distinct signatures vs. size.

We assessed the size distribution based on both total abundances and distinct sequences (Figure 1). For total abundance, approximately 90.1% small RNAs were 20-24 nt in length, with 21 and 24 nt being the major size classes (Figure 1a). For the proportion of nonredundant sequence of each size, we found that 24 nt sequences are prevailing in all the libraries, while the 21 nt sequence is less abundant (Figure 1b). Further analysis revealed that the distribution of nonredundant sRNAs for various size classes was similar among the six libraries, but the size distribution of redundant sRNAs was greatly different for *Egt *infection and heat stress treatment. Both JD8 and JD8-*Pm30 *showed a similar pattern of 21-nt small RNA reduction and 24-nt small RNA increase in abundance after inoculation with *Egt *for 12 hours (Figure 1a). However, for the library for high temperature treatment both the 21 and 24-nt small RNAs were reduced in abundance (Figure 1a). The canonical miRNAs are 21 nt while canonical heterochromatic siRNAs are 24 nt. These observations indicated that the expression of miRNAs and siRNAs significantly altered after *Egt *infection and heat stress, suggesting that miRNAs and siRNAs could be involved in the extensive regulation of gene expression in response to powdery mildew infection and heat stress in wheat.

### Identification of known miRNAs

We searched for known miRNAs in the six small RNA libraries by use of homolog analysis to find miRNA sequences (orthologs/paralogs) matching at least 18 nt and leaving 3 nt for possible sequence variations. A total of 72 known miRNAs from 51 families were identified (Additional files 1 and 2). Our previous miRNA cloning by 454 sequencing in wheat have identified 43 miRNA families [38], among which 37 miRNA families were confirmed in current Solexa data set, however, other 6 families, including TamiR479, TamiR503, TamiR508, TamiR513, TamiR518, and TamiR522, were absent in our current Solexa data set. This is probably due to that the small RNA library for 454 sequencing was constructed from the pooled wheat RNA isolated from leaves, roots and spikes. In addition, 14 miRNA families, which were not found in the previous 454 sequencing data, were identified in present study with lower frequency. This indicated that these miRNAs were not abundant in wheat and could be detected only in the larger sequencing data set. Seven known rice miRNAs, including miR528, miR530, miR535, miR812, miR818, miR820, and miR827, which were not found in other species like *Arabidopsis*, were also represented in our data set, suggesting that they might belong to monocot-specific miRNAs.

### Identification of novel wheat miRNAs

For the identification of novel wheat miRNAs, we firstly rely on wheat EST sequences as miRNA surrounding sequences in prediction, since the information of wheat genome sequence is limited. We found that a total of 330,925 small RNA sequences can be perfectly matched to at least one EST. The matched ESTs were then searched against Rfam and protein database to eliminate non-coding RNAs such as rRNA and tRNA as well as the degradation products from protein-coding sequences. With this analysis, we obtained 38167 sequences which are used to predict for fold-back RNA secondary structure. And we evaluated reads that fell within potential miRNA-like hairpins, considering the following five criteria: (1) the pairing characteristics of the hairpin; (2) the expression of the candidate, as measured by the abundance of sequence reads sharing the same 5' terminus; 3) presence in no less than two independent libraries, 4) the presence of miRNA* for several new microRNAs; 5) the absence of annotation suggesting non-miRNA biogenesis. These hairpins were further checked manually to ensure that they were accorded with the new criteria for annotation of plant miRNAs, provided recently by Meyers et al [39]. Based on such analysis, we identified a total of 81 novel microRNA candidates (Additional files 3, 4 and 5). The length of the newly identified miRNAs range from 21 bp and 24 bp in length, and the negative folding free energies vary from -98.4 to -34.5 kcal mol^-1 ^(with an average of -59.5 kcal mol^-1^) according to MFOLD, which is similar to the free energy values of other plant miRNA precursors (-72.4 kcal mol^-1 ^in wheat, -71.0 kcal mol^-1 ^in rice and -59.5 kcal mol^-1 ^in *Arabidopsis *respectively). We also found the presence of 17 miRNA* in our libraries. Moreover, we took advantage of approximate 4× depth genomic sequences of *Brachypodium distachyon*, a grass species related to wheat, for further discovery of novel miRNAs. A total of 20 novel miRNAs were identified (Additional file 6), among which 16 (Additional files 6 and Additional file 4, underlined) can not be matched to wheat EST sequences due to the limitation of available wheat EST sequences.

In order to understand the conservation of new identified wheat miRNA, we compared wheat novel miRNAs to genomes of species representing important lineages, including maize, *Arabidopsis*, rice, *Brachypodium distachyon *and soybean. Among the 81 new miRNAs, 67 were wheat specific, and the others were conserved in different species. For example, miR2019 was conserved in monocots such as wheat, *Brachypodium distachyon*, rice and maize, suggesting that it might be monocot-specific miRNA. miR2072 and miR2050 were conserved in *Brachypodium distachyon*, wheat and rice; miR2070 was conserved in *Brachypodium distachyon *and wheat, miR2014 was conserved in rice and wheat.

**Figure 2:**
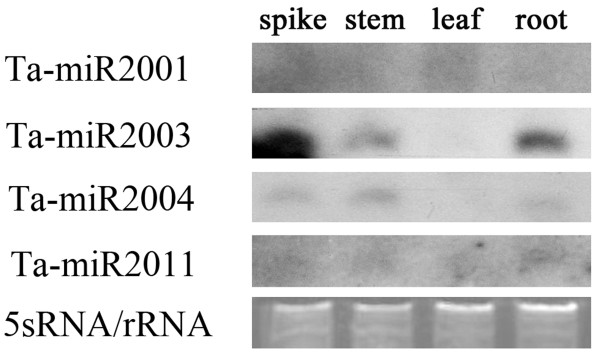
**Expression patterns of novel miRNAs in wheat**. RNA gel blots of low molecular weight RNA from different tissues, including spikes, stems, leaves and roots, were probed with labeled oligonucleotides. The tRNA and 5 S RNA bands were visualized by ethidium bromide staining of polyacrylamide gels and served as loading controls.

Northern detection represents a useful criterion for authenticating miRNAs [40]. In order to determine the expression patterns of these novel wheat miRNAs, we analyzed the expression levels of novel miRNAs in leaf, stem, spike and root tissues by Northern blot. A total of 10 novel miRNAs with high frequency in Solexa sequencing were tested, and 4 showed the obvious signals in Northern blot analysis (Figure 2). The results revealed that miR2003 and miR2004 were more abundant in stems, spikes and roots than in leaves, while miR2001 and miR2011 showed a little more expression level in leaves and roots, respectively.

A total of 149 genes were predicted as potential targets for these 81 new identified miRNAs (Additional file 7). Up to 62 new identified wheat miRNAs have predicted targets, whereas 10 miRNAs do not, which could be due to limitation of wheat EST sequences. We found that 52 new miRNAs have 139 targets with definite function, among which 16 encode proteins responding to disease or stress, 6 for transcription factor, 117 encode other proteins and most of them are involved in cellular metabolism. This is quite different as compared to the conserved miRNAs, as most of their targets are transcription factors. Interesting, we found that 28 target genes were putatively regulated by more than 2 miRNAs. It has also been widely suggested that microRNAs, similarly to transcription factors, can act in combination (or cooperatively) by binding to the same mRNA in a concentration-dependent manner [41].

### Response of wheat miRNAs to powdery mildew infection and heat stress

**Figure 3:**
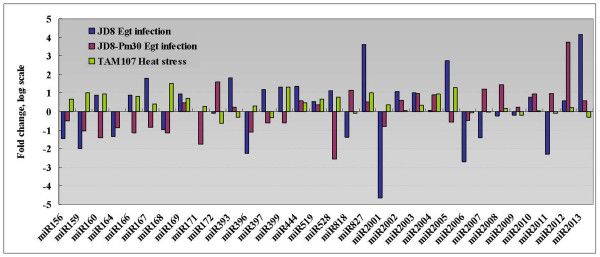
**Expression profiling of MiRNA families using high-throughput sequencing**. Blue and red bars represented the JD8 and JD8-*Pm30 *at 12 hr post-inoculation with *Egt *relative to uninoculated leaves. Green bar represented the TAM107 after the 40°C for 1 h relative to un-inoculated leaves.

**Figure 4:**
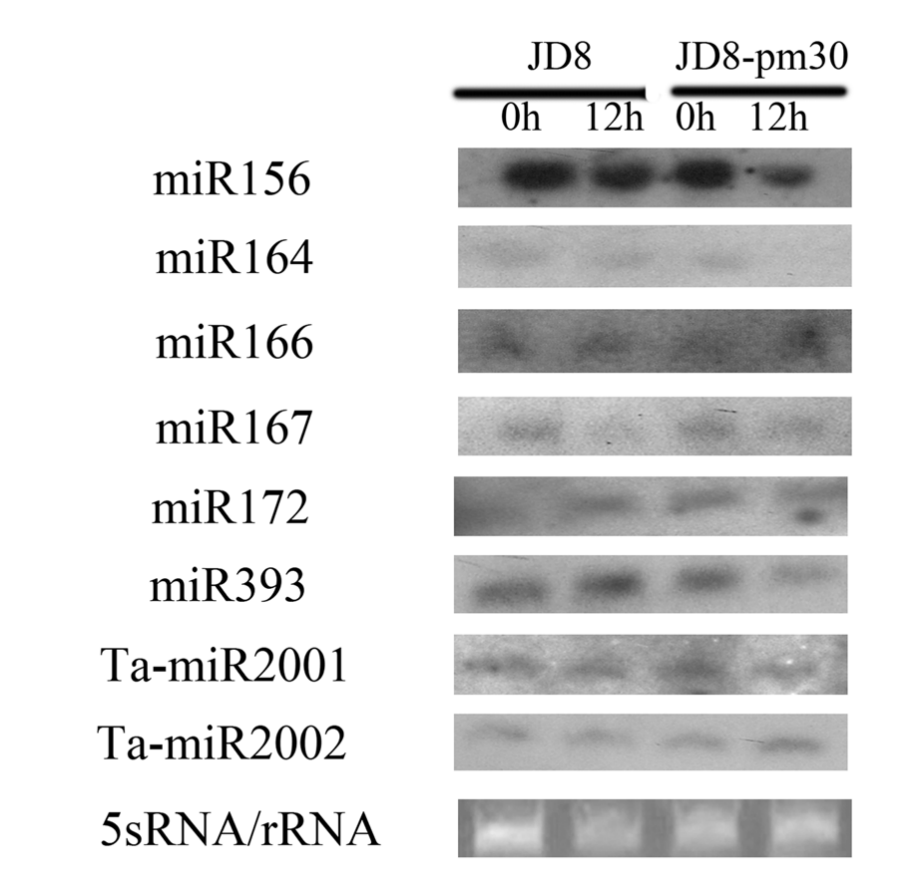
**Powdery mildew infection responsive expression of wheat miRNAs by Northern blot**. The tRNA and 5 S RNA bands were visualized by ethidium bromide staining of polyacrylamide gels and served as loading controls. Disease susceptible wheat cultivar Jingdong8 (JD8) and its near-isogenic line Jingdong8-*Pm30 *(JD8-*Pm30*) having a resistant gene *Pm30 *were used. "0 h" means the uninoculated leaves and "12 h" means the leaves were inoculated by the *Erysiphe graminis f. sp. tritici *(*Egt*) (Isolate E09).

It has been reported that high-throughput sequencing can be used as a tool for miRNA expression profiling [42]. Therefore, in this study expression profiling of some known and new identified miRNAs was also determined based on Solexa sequencing data (Additional files 1, 2, 3, and 4). The miRNA frequency was normalized in "transcripts per million (TPM) " for each library and we found that the top 10 abundant sequences are deeply conserved miRNAs. We noted that miR168a, whose abundance relative to the total number of miRNAs can reached to 48%, was the most abundant one (Additional files 2). Previous study also showed that miR168a was also the most abundant miRNA in rice in high-throughput sequencing [43,44]. Compared to the conserved miRNAs, most of the novel miRNAs were relatively in low abundance as indicated by their frequencies (Additional files 4). Among the 81 novel miRNAs, only 13 (18%) had at least 20 transcripts per million (TPM) in one library with the highest abundance 586 TPM for miR2005. Further analysis revealed that among the 132 miRNA families represented in present study, 43 miRNAs had at least 20 transcripts per million (TPM) in at least one library (Additional files 2 and 4). Firstly, in order to determine the response of wheat miRNAs to powdery mildew infection, we compared the TPM value changes before and after infection, and looked for the miRNAs that were up- or down- regulated after powdery mildew infection in JD8 and JD8-*Pm30 *as compared to the controls (Figure 3). By statistical analysis according to Poisson distribution, a total of 22 miRNAs were identified to be significantly responsive to powdery mildew infection in at least one genotype (P < 0.05). We classified these miRNAs into 3 groups according to their expression patterns in different genotypes, that is, JD8 specific responsive miRNAs (Group 1), JD8-*Pm30 *specific responsive miRNAs (Group 2), miRNAs responsive in both JD8 and JD8-*Pm30 *(Group 3). Group 1 contained 8 miRNAs, among which, 3 (miR2001, miR2006, miR2011) were decreased, and 5 others (miR393, miR444, miR827, miR2005 and miR2013) were increased after powdery mildew infection in JD8. miR2013, miR827 and miR2001 showed the highest expression alteration with 18, 12, and -25 fold changes. In Group 2, miR171 was decreased, and 2 others (miR2008 and miR2012) were increased after powdery mildew infection. Group 3 contained 10 miRNAs, in which miR156, miR159, miR164 and miR396 were significantly decreased with same expression pattern in JD8 and JD8-*Pm30*. The others exhibited the opposite expression pattern in JD8 and JD8-*Pm30*. To further characterize the expression of some miRNAs that showed significantly alteration in Solexa deep sequencing, we performed northern blot analysis for eight miRNAs (Figure 4). It was found that after powdery mildew infection miR156 was down-regulated both in JD8 and JD8-Pm30, miR164 was down-regulated only in JD8-pm30 but not in JD8, miR393 was down-regulated only in JD8-pm30 but not in JD8 (Figure 4).

**Figure 5:**
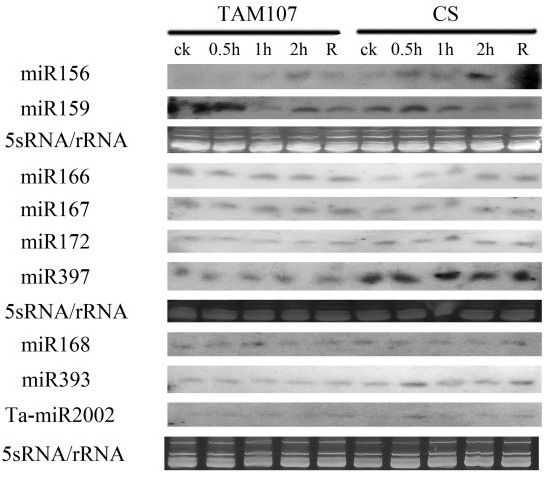
**heat stress responsive expression of wheat miRNAs by Northern blot**. The tRNA and 5 S RNA bands were visualized by ethidium bromide staining of polyacrylamide gels and served as loading controls. Heat tolerant cultivar TAM107 and heat susceptible cultivar CS were treated by heat treatment for 0.5, 1, 2 hours and were returned to normal growth condition (R).

Secondly, in order to identify heat responsive miRNAs, we compared normalized expression profiles of miRNA families in TAM107 before and after heat stress (Figure 3). Among the 32 miRNA families, 9 were putatively heat responsive according to the statistical analysis (P < 0.05). For example, miR172 was significantly decreased with 1.5 fold changes, and 8 miRNAs, including miR156, miR159, miR160, miR166, miR168, miR169, miR827, and miR2005, were up-regulated with the highest expression change of 2.9 fold for miR168. We further performed the Northern blot analysis to determine the expression patterns of 9 miRNAs in heat tolerant genotype TAM107 and heat susceptible genotype Chinese Spring (CS) after heat treatment for 0.5, 1, 2 hours and returning to normal growth condition (Figure 5). The results revealed that expression of miR156 was up-regulated in both TAM107 and CS genotypes after heat treatment, miR159 was down-regulated only in CS genotype after 2 h heat treatment, miR166 was up-regulated only in CS genotype after 2 h heat treatment, miR393 and Ta-miR2002 were up-regulated only in CS genotype after 0.5 h heat treatment (Figure 5). It was also noted that expression of some miRNAs, such as miR156 and miR393 were significantly changed at 0.5 h after heat treatment, suggesting that the expression of some miRNA are responsive to the vary short heat treatment.

Thirdly, we identified that 9 miRNAs are co-regulated by both powdery mildew infection and heat stress (Figure 3), among which two (miR827, miR2005) were up-regulated both in powdery mildew infection and heat stress, indicating that they might play important roles in both abiotic and biotic stress response in wheat. The others exhibited opposite expression pattern in response to powdery mildew infection and heat stress.

**Figure 6:**
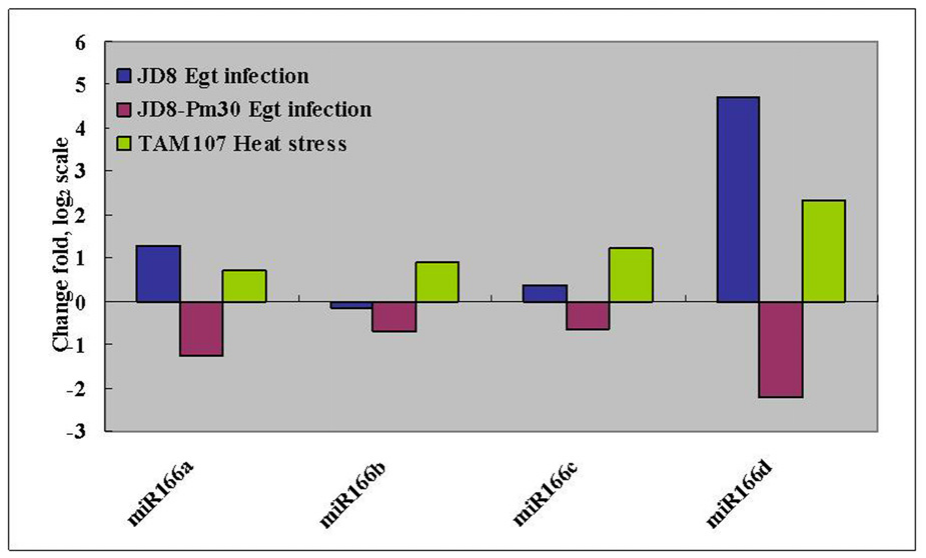
**Different members in miRNA family miR166 represented the different expression patterns**.

In addition, we also found that the frequency of different members in one miRNA family varied greatly, suggesting the functional divergence within families. It is thus important to examine which members of miRNA family are more responsive to powdery mildew infection and heat stress. We found that one miRNA family in which different members have different expression patterns (Figure 6). For example, the four members of miR166 exhibited the different expression pattern in response to stress. MiR166a and miR166 d were significantly altered after powdery mildew infection, but miR166c and miR166 d were not. For heat stress, only miR166 d were significantly altered. We also found 8 cases (miR156, miR159, miR172, miR167, miR169, miR396, miR399 and miR818) where all the members of a miRNA family were expressed at similar pattern in response to powdery mildew infection or heat stress.

### Expression patterns of potential target genes in response to powdery mildew infection and heat stress

**Figure 7:**
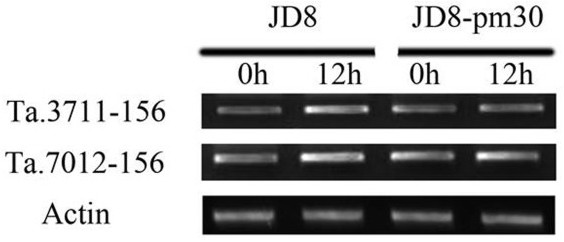
**Semi-quantitative RT-PCR analysis of putative target genes of miR156 in response to powdery mildew infection**. Disease susceptible wheat cultivar Jingdong8 (JD8) and its near-isogenic line Jingdong8-*Pm30 *(JD8-*Pm30*) having a resistant gene *Pm30 *were used. "0 h" means the uninoculated leaves and "12 h" refers to the leaves were inoculated by the *Erysiphe graminis f. sp. tritici *(*Egt*) (Isolate E09).

The differential expression of a miRNA is expected to have opposite effects on its target gene(s) expression. In this study, we further performed semi-quantitative RT-PCR analysis to determine the expression patterns of 2 putative target genes of miR156 (Figure 7), which is responsive to powdery mildew infection (Figure 4). Negative correlations were observed between miR156 and its putative target genes Ta3711 and Ta7012. In accordance with solexa sequencing, miR156 was down-regulated in northern blot after powdery mildew infection both in JD8 and JD8-Pm30, while their putative targets gene Ta3711 and Ta7012 were up-regulated, respectively. Then, we tested if the miRNA directs the cleavage of putative mRNAs using an Agrobacterium-mediated delivery system to co-express miR156 and Ta3711 mRNA in *N. benthamiana *leaf tissue. Four constructs (35S::miR156, 35S::Ta3711, 35S::mTa3711) were inoculated and expressed. Co-expression of 35S::Ta3711 with 35S::miR156 resulted in loss of the full length Ta3711 transcript form, and co-expression of 35S::mTa3711 (encoding a Ta3711 transcript that carries an altered miR156 binding site; see Materials and methods) with 35S::miR156 resulted in detectable transcripts of the Ta3711 size (Additional file 8). The results indicated that miR156 directed the cleavage of Ta3711.

**Figure 8:**
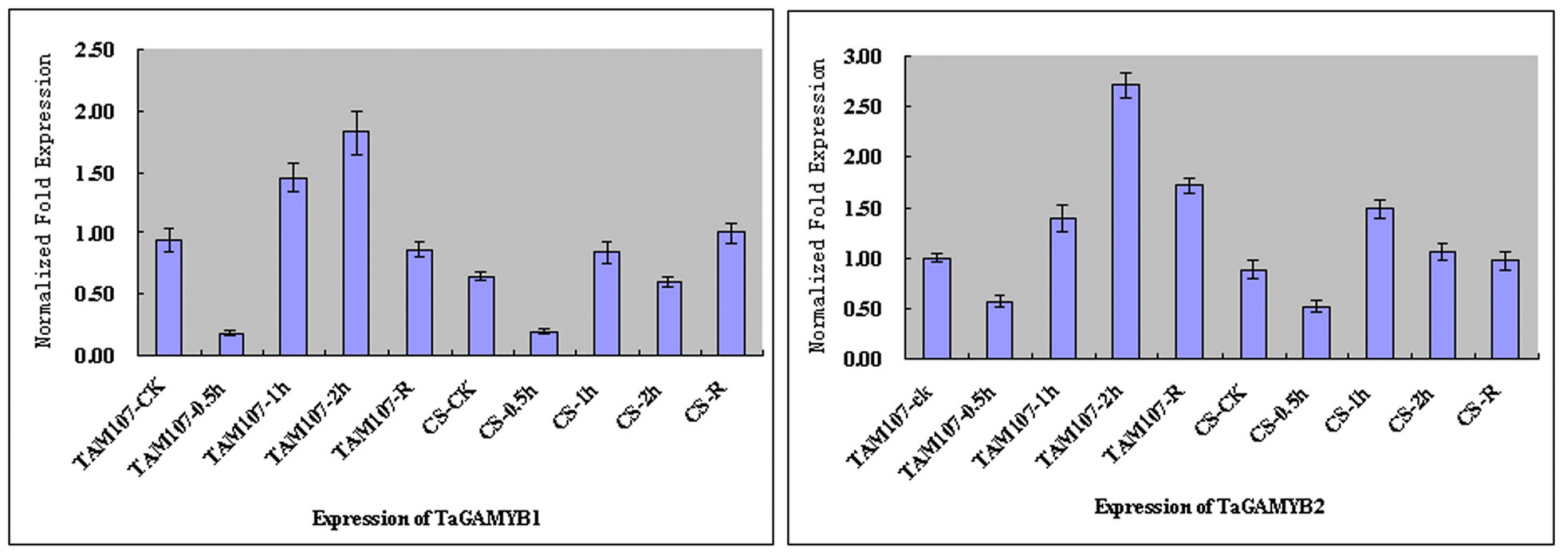
**Realtime-PCR analysis of *TaGAMYB1 *and *TaGAMYB2 *response to heat stress**. Heat tolerant cultivar TAM107 and heat susceptible cultivar CS were treated by heat treatment for 0.5, 1, 2 hours and were returned to normal growth condition (R).

Our previous study had identified a number of heat responsive transcripts in TAM107 treated at 40°C for 1 hour by using GeneChip Wheat Genome Array [7]. In this study, by realtime PCR, we determined the expression of *TaGAMYB1 *(UniGene NO.Ta24098) and *TaGAMYB2 *(UniGene NO.Ta138051) targeted by miR159 in heat tolerant genotype TAM107 and heat susceptible genotype CS after heat treatment for 0.5, 1, 2 hours and returning to normal growth condition (Figure 8). The results indicated that expression of both *TaGAMYB1 *and *TaGAMYB2 *were significantly decreased after heat treatment for 0.5 hours and then increased after 1 and 2 hours (Figure 8), which was opposite to the expression of miR159.

## Discussion and Conclusions

Identification of entire set of miRNAs and their targets will lay the foundation to unravel the complex miRNA-mediated regulatory networks controlling development and other physiological processes. Several computational studies estimated that organisms probably contain about 1% miRNA genes of the total protein-coding genes [34,35,45]. Recently, a large number of miRNA have been found in various species. For example, the identified number of miRNA in *Arabidopsis*, rice and maize were 187, 353 and 96, respectively. A total of 58 wheat miRNAs were identified by 454 sequencing, [38]. In present study, our Solexa high-throughput sequencing of wheat small RNAs revealed a diverse and complex small RNA population and expression of the 51 known plant miRNA families (Additional files 1 and 2) and 81 novel miRNAs (Additional files 3 and 4) were determined. This increased the number of known wheat miRNA families from 43 to 132. Although the number of deeply conserved miRNA families in wheat largely remains the same as in *Arabidopsis*, all of the newly identified wheat miRNAs do not appear to be conserved in *Arabidopsis *and have predicted target genes with more diverse functions than those of conserved miRNAs. Recent deep sequencing of plant small RNA libraries also demonstrated that plants express more non-conserved than conserved miRNAs [8,42,46]. Only 5 miRNAs were conserved in different species, suggesting that wheat appears to have evolved species-specific miRNAs because of the functional diversification among the various species. Therefore, our study revealed that wheat genome encoded more non-conserved miRNA families than conserved miRNA families. It has been proposed that these non-conserved miRNAs presumably emerged and dissipated in short evolutionary time scales [42,47], and such rapid emergence of new genes is likely facilitated by the small size and simple architecture of miRNA genes [47,48].

By using Northern blotting, at least 3 and 5 wheat miRNAs families were found to be responsive to powdery mildew infection and heat stress, respectively. It is interesting to note that the members of the same miRNA family were differentially regulated in the response to powdery mildew infection and heat stress. This is consistent with results obtained from cold-stressed *Populus *[30], drought-stressed rice [49] and UV-B-treated *Arabidopsis *[50]. Therefore, the functions of plant miRNAs can be dissimilar even if they share a high degree of sequence similarity and belong to the same family [30]. In addition, several reported stress-responsive miRNAs in *Arabidopsis *and *Populus *are also involved in wheat response to powdery mildew infection and heat stress. For example, miR156 and miR160 were significantly repressed in the galled loblolly pine stem infected with the fungus *C. quercuum f.sp.fusiforme *[32], and miR156, miR164 and miR160 were induced in tobacco after plant virus's infection [51]. In *Arabidopsis*, miR156 and miR164 were induced by infection with plant virus TYMV p69, and also induced in transgenic *Arabidopsis *plants expressing the viral silencing suppressor P1/HC-Pro [52]. In this study, we also found that miR156 was significantly repressed by powdery mildew infection, whereas, miR156 was induced in response to heat stress. It was reported that miR393 was induced by a bacterial PAMP peptide flg22 at 20 to 60 min post-treatment [31]. And in our study, expression of miR393 was also strongly increased in JD8 under infection of *Egt*. Moreover, we also found that 8 and 2 novel wheat miRNAs families were responsive to powdery mildew infection and heat stress. Taken together, these stress-related miRNAs identified in this study may function in one of the most critical defense systems for wheat biotic and abiotic stress tolerance, but the underlying molecular mechanism remains an area to be elucidated.

Auxin is an important hormone in plants, and involved in many plant processes including cell elongation and thus growth. Report shows that susceptible barley cultivar infected by powdery mildew have a remarkably increase of IAA level after inoculation [53] and simultaneously, IAA level decreased after the inoculation by parasite in the resistant barley cultivar "Professor Schiemann" [53]. More recently, it was reported that miR393 was involved in auxin signaling pathway and played an important role in basal defense. Previous studies revealed that miR160, miR166, miR167 and miR393 may involve in auxin signaling pathway by regulating different transcription factors. One interesting observation in this study is that the expression patterns of miR393 related to auxin signaling pathway was significantly different between near isogenic JD8 and JD8-Pm30. Therefore, we speculated that auxin pathways affected by miRNAs could play important roles in powdery mildew disease resistance.

## Methods

### Plant materials

Hexaploid wheat (*Triticum aestivum *L.) line JD8 (susceptible to wheat powdery mildew) and its near-isogenic resistant line JD8-*Pm30 *were grown in a growth chamber at a relative humidity of 75% and 26/20°C day and night temperature with light intensity of 3000 lx. Seven-day-old plants were used for all experiments. One isolate of the powdery mildew fungus *Erysiphe graminis f. sp. tritici *(*Egt*) (Isolate E09) was maintained on the wheat cultivar Fidel by weekly transfer to new plants. Inoculations were performed at a density of 100-150 conidia/mm2 by brushing plants. Leaves were collected at 12 hours after infection.

Two wheat genotypes, heat susceptible 'Chinese Spring' (CS) and heat tolerant 'TAM107' were used in this study. Seeds were surface-sterilized in 1% sodium hypochlorite for 15 min, rinsed in distilled water, and soaked in dark overnight at room temperature. The germinated seeds were transferred into the pots (25 seedlings per pot) containing vermiculite. The seedlings were subjected to 40°C (heat stress) for 0.5, 1 and 2 hours and returned to normal conditions for 2 hours. At the end of heat treatments, the leaves of the seedlings were frozen immediately in the liquid nitrogen, and stored at -80°C for further use.

### RNA extraction and small RNA cloning

Total RNA was isolated from the frozen leaves by using Trizol agent (TaKaRa, Inc., Dalian, China) according to the manufacturer's instructions. Cloning of the small RNAs was performed as described by Sunkar and Zhu [23]. Briefly, low molecular weight RNA was enriched by 0.5 M NaCl and 10% PEG8000 precipitation. About 100 μg low molecular weight RNA was separated on a denaturing 15% polyacrylamide gel. Labeled RNA oligonucleotides with 18 and 26 nt were used as size standards. The nucleotides from 18 to 26 nt were excised, and RNA was eluted overnight with 0.4 M NaCl at 4°C. The RNA was dephosphorylated by alkaline phosphatase (New England Biolabs Inc, Beijing China) and recovered by ethanol precipitated. The small RNAs were then ligated sequentially to RNA/DNA chimeric oligonucleotide adapters, and then reverse transcription was preformed, followed by PCR amplification. The resulting PCR products were sequenced using Solexa technology.

### Data analysis

Automated base calling of the raw sequence and vector removal were performed with PHRED and CROSS MATCH programs [23,54]. Trimmed 3' and 5' adapters sequences, removed RNAs less than 17 nt and polyA, only sequences longer than 17 nt with a unique ID were used for further analysis. These sequences were used to search for the Rfam database [55] with BLASTN [56] to remove most non-siRNA and non-miRNA sequences. Putative origins for the remaining sequences were identified by BLASTN search against wheat EST database from NCBI. The protein-coding EST sequences were removed and the remaining non-coding candidate wheat ESTs with perfect matches with small RNA sequences were used for fold back secondary structure prediction with MFOLD program [57]. In NCBI Unigene database, closely related wheat ESTs have been assembled to Unigene cluster, therefore the Unigene accessions were selected and recorded. Based on these analyses, putative miRNAs were then searched against NCBI NT database to check whether these miRNAs exist in other species. We also map the small RNA sequences to *Brachypodium distachyon *genomic sequences, which were downloaded from http://www.brachypodium.org/.

Target gene prediction was carried out as described by Jones-Rhoades et al [19,58]. It was performed by searching the wheat EST database and NCBI NT database for miRNA complementary sequences. These criteria include allowing one mismatch in the region complementary to nucleotide positions 2 to 12 of the miRNA but not at the position 10/11 which is predicted cleavage site, and three additional mismatches were permitted between 12 and 22 nucleotide positions, but no more than two continuous mismatches within this region.

### Differential expression analysis of miRNAs based on high-throughput sequencing

The frequency of miRNA was normalized by total number of miRNAs in every sample. The fold change between treatment and control was calculated as: Foldchange = log2(treatment/control). And then statistically analysis was performed according to Poisson distribution. The P-value was calculated based on the formula

The miRNAs with TPM less than 20 showed the less expression level, which did not be included for the differential expression analysis.

### RNA gel blot analysis

Low molecular weight RNA was loaded and resolved on a denaturing 15% polyacrylamide gel, and transferred electrophoretically to Hybond-N+ membranes (Amersham Biosciences, Buckinghamshire, UK). Membranes were UV cross-linked and baked for 2 hours at 80°C. DNA oligonucleotides complementary to miRNA sequences were end labeled with γ-^32^P-ATP using T4 polynucleotide kinase (TaKaRa, Dalian, China). Membranes were prehybridized for more than 8 hours and hybridized overnight using Church buffer at 38°C. Blots were washed three times (two times with 2 × SSC + 1% SDS and one time with 1 × SSC + 0.5% SDS) at 50°C. The membranes were briefly air dried and then exposed to X-ray films for autograph at -80°C.

### Infiltration of Agrobacterium tumefaciens intoN. Benthamiana

Precursor sequences of miR156 including the hairpin structures, the Ta3711 and mTa3711 were cloned to downstream of 35 S promoter, respectively. A mutated version of the Ta3711 transgene (mTa3711) was generated by PCR. The mutated mTa3711 primers used were as follows: 5'-ATCTTCAGCACGCACTGTCACTACTCTCTAGCAACCCAGTGG-3 'and 5'-CCACTGGGTTGCTAGAGAGTAGTGACAGTGCGTGCTGAAGAT-3'. The nucleotide sequence in miR156 binding domain of the mTa3711 was changed from CATGCTCTCTCTCTTCTGTCA to CACGCACTGTCACTACTCTCT.

35S::miR156, 35S::Ta3711, 35S::mTa3711 were introduced into A. tumefaciens and the bacteria injected into N. benthamiana leaves with a syringe according to the method of Llave [59]. For co-injections of two different constructs, bacteria were resuspended in infiltration medium (0.5' Murashige and Skoog salts, 5% sucrose, 0.5 g/l Mes) at OD600 = 1, and incubated for 3 hours at room temperature with 150 mM acetosyringone. Zones of infiltration were harvested at 4 and 5 days after injection for RNA isolations.

### Semi-quantitative RT-PCR analysis

Total RNA was isolated by using Trizol (Invitrogen, USA) according to the manufacturer's instructions and treated with RNase-free DNase I (Promega, Madison, USA). Two microgram of total RNA from each sample was used for first-strand cDNA synthesis in 20 μl reactions containing 50 mM Tris-HCl (pH 8.3), 75 mM KCl, 3 mM MgCl_2_, 10 mM DTT, 50 μM dNTPs, 200 U M-MLV reverse transcriptase (Promega, Madison, USA) and 50 pmol oligonucleotides T15. Reverse transcription was performed at 37°C for 60 min with a final denaturation at 95°C for 5 min. Gene-specific RT-PCR primers for 5 miRNA targets were designed according to the EST sequences.

Three RT-PCR replications were conducted using independently isolated total RNAs with the following thermal cycling parameters: 94°C for 30 sec, 57°C for 30 sec, and 72°C for 30 sec. A 350 bp β-actin gene fragment was amplified as a positive control using the primer pair 5'-CAGCAACTGGGATGATATGG-3' and 5'-ATTTCGCTTTCAGCAGTGGT-3'. The RT-PCR products were sequenced to verify the specificity of PCR amplifications. The primers used for RT-PCR were listed in the Additional file 9.

### Realtime PCR analysis

The *TaGAMYB1 *gene specific primer was designed using DNAMAN software as followings: 5'-CCTGAATTGAGCGACACC' and 5'-ACACACTCCGACTT-CACTG-3'. The *TaGAMYB2 *gene specific primer was designed using DNAMAN software as followings: 5'-GTGGGGCGATTTCATTGAT-3' and 5'-TTGAGTA-TGCGGACCAGTTG-3'. PCR reactions were performed in a volume of 20 μl containing 10 pmol primers 10 mmol/l Tris-HCl)pH8.5), 50 mmol/l KCl, 2 mmol/l MgCl2, 0.4 μl DMSO, 200 mmol/l dNTPs, 1 U Taq DNA Polymerase (TaKaRa, Dalian), 0.5 μl SYBR GREEN I. PCR amplification protocol was followed by 95°C (3 min) and 40 cycles of amplification cycle (95°C (30 s), 55°C (30 s), and 72°C (1 min)) using Opticon PTC200 system (MJ Research, USA).

All reactions were run in triplicate and included no template and no reverse transcription controls. Quantification results were expressed in terms of the cycle threshold (CT) value according to the baseline adjusted to 0.04. The comparative CT method (PE Applied Biosystems) was used to quantify relative gene expression compared with actin. Briefly, the CT values were averaged for each triplicate. Differences between the mean CT values of Ta*GAMYB *and those of *β-actin *were calculated as ΔCTsample = CT Ta*GAMYB *- CT*β-actin*. Final results, the sample relative expression level were determined as 2 -ΔCtsample. Statistical significance was tested using the Student's t-test (P < 0.05).

## Authors' contributions

MX, YW and YY carried out the small RNA cloning and data analysis. CX, HP and ZN prepared the materials and performed the RNA gel blot analysis and semi-quantitative RT-PCR analysis. MX and YW performed the experiments of the infiltration of Agrobacterium tumefaciens into *N. Benthamiana *and realtime PCR analysis. QS designed the experiments and wrote the manuscript together with YY, ZN and CX. All authors read and approved the final manuscript.

## Supplementary Material

Additional file 1 **Expression changes of known miRNA in response to wheat powdery mildew infection and heat stress**. Click here for file

Additional file 2 **Known miRNA expression in response to wheat powdery mildew infection and heat stress**. Click here for file

Additional file 3 **Fold changes of new miRNA in response to wheat powdery mildew infection and heat stress**. Click here for file

Additional file 4 **New identified candidate miRNAs in wheat**. Click here for file

Additional file 5 **The putative hairpin structures of new identified miRNAs**. Click here for file

Additional file 6 **New identified miRNAs by 4× depth *Brachypodium distachyon *genomic sequences**. Click here for file

Additional file 7 **The putative targets of predicted miRNAs**. Click here for file

Additional file 8 **Figure S1 MiR156 directs the cleavage of Ta3711 transcripts**. Click here for file

Additional file 9 **Primer sequences of miRNA target genes used for RT-PCR analysis**. Click here for file
