# Supplementary material for: Diverse set of microRNAs are responsive to powdery mildew infection and heat stress in wheat (Triticum aestivum L.)
Source: BMC Plant Biol. 2010 Jun 24;10:123. doi: 10.1186/1471-2229-10-123 (PMC3095282; doi:10.1186/1471-2229-10-123)
Supplement: Additional file 1 — Expression changes of known miRNA in response to wheat powdery mildew infection and heat stress. [file 1471-2229-10-123-S1.DOC]

Additional file 1 Expression changes of known miRNA in response to wheat powdery mildew infection and heat stress

| Known miRNA | sequence | Fold change(log2 JD8-*Egt*/JD-CK) | Fold change(log2 JD8-*Pm30-Egt*/JD*-Pm30*-CK) | Fold-change(log2 TAM107-Heat/TAM107-CK) |
| --- | --- | --- | --- | --- |
| miR156a | TGACAGAAGAGAGTGAGCAC | -1.44** | -0.52** | 0.65** |
| miR156b | TGACAGAAGAGAGCGAGCAC | -2.34** | -0.01** | 0.90** |
| miR156c | TGACAGAAGAGAGAGAGCAC | -1.79** | -0.08** | 0.80* |
| miR156d | TGACAGAAGAGAGTGAGCA | -1.52 | -0.71 | 0.55** |
| miR156e | TGACAGAAGAGAGTGAGCACT | -0.75 | -0.62 | 0.82 |
| miR156f | TGACAGAAGAGCGTGAGCAC | -1.38** | -0.05* | 1.25* |
| miR156g | GACAGAAGAGAGTGAGCAC | -0.83* | -1.36** | 0.82 |
| miR159a | TTTGGATTGAAGGGAGCTCTG | -2.053** | -1.15 | 1.18** |
| miR159b | TTTGGATTGAAGGGAGCTCTGA | -1.77** | -0.84 | 1.00 |
| miR160 | TGCCTGGCTCCCTGTATGCCA | 0.88* | -1.43 | 0.80* |
| miR164 | TGGAGAAGCAGGGCACGTGCA | -1.28** | -1.02** | -0.25** |
| miR166a | TCGGACCAGGCTTCATTCCCC | 1.29** | -1.27** | 0.71** |
| miR166b | TTCGGACCAGGCTTCATTCCC | -0.16 | -0.70 | 0.90 |
| miR166c | TCGGACCAGGCTTCATTCCCT | 0.37** | -0.67 | 1.22 |
| miR166d | TCGGACCAGGCTTCATCCCCC | 4.70** | -2.23** | 2.33** |
| miR167a | TGAAGCTGCCAGCATGATCTA | 2.03** | -0.63 | 0.51** |
| miR167b | TGAAGCTGCCAGCATGATC | 1.63** | -0.91** | -0.28** |
| miR167c | TGAAGCTGCCAGCATGATCTG | 1.82** | -0.66 | 0.63** |
| miR168 | CGCTTGGTGCAGATCGGGAC | -0.45** | -0.83** | 1.52** |
| miR169a | CAGCCAAGGATGACTTGCCGA | 1.30** | 0.37** | 0.66** |
| miR169b | AGCCAAGGATGACTTGCCGGC | 1.13** | 0.49** | 0.68* |
| miR169c | CAGCCAAGGATGACTTGCCGGA | 0.79 | 0.32 | 0.97 |
| miR169d | TAGCCAAGGATGACTTGCCTG | 0.16 | 0.90 | 0.91 |
| miR171 | TGATTGAGCCGCGCCAATATC | -0.01 | -1.75* | 0.26** |
| miR172a | AGAATCTTGATGATGCTGCAT | -0.08** | 1.60** | -0.63** |
| miR172b | AGAATCTTGATGATGCTGCA | 0.24 | 0.81* | -1.04** |
| miR393a | TTCCAAAGGGATCGCATTGAT | 1.86** | 0.27 | -0.07** |
| miR393b | TCCAAAGGGATCGCATTGATC | 0.87* | -1.01 | 0.29* |
| miR396a | TCCACAGGCTTTCTTGAACTG | -2.27** | -1.10 | 0.36 |
| miR396b | TTCCACAGCTTTCTTGAACTT | -2.73 | -1.09 | -0.24** |
| miR397 | TTGAGTGCAGCGTTGATGAAC | － | 0.64 | -0.32** |
| miR399a | TGCCAAAGGAGAATTGCCCTG | 1.15 | 1.06 | 1.57 |
| miR399b | TGCCAAAGGAGAGTTGCCCTG | 1.39 | -0.82 | 1.09 |
| miR444 | TGCAGTTGCTGCCTCAAGCTT | 1.35* | 0.57 | 0.46** |
| miR818a | TTCTTATATTATGGGACGGAG | -1.43* | 1.21** | -0.04** |
| miR818b | TCTTATATTATGGGACGGAGG | -2.70** | -0.46 | -0.08** |
| miR818c | CTCTTATATTATGGGACGGAG | -1.23 | 0.88 | -0.38** |
| miR827 | TTAGATGACCATCAGCAAACA | 3.61* | 0.52 | 0.99* |
| miR519 | TTCGGAATTACTTGTCGCAGA | 0.54 | 0.36 | － |
| miR528 | TGGAAGGGGCATGCAGAGGAG | 1.13** | -2.56* | 0.31 |
| miR319 | TGGACTGAAGGGAGCTCCCT | － | － | 0.69 |
| miR390 | AAGCTCAGGAGGGATAGCGC | － | － | -0.58 |
| miR394 | GGCATTCTGTCCACCTCC | － | － | － |
| miR395 | TGAAGTGTTTGGGGGAACTC | -1.30 | － | － |
| miR408 | TGCACTGCCTCTTCCCTGGC | － | － | － |
| miR827 | TTAGATGACCATCAACAAACA | 3.62** | 0.52 | 0.99* |
| miR845 | TGCTCTGATACCAATTGATGGCAC | － | － | － |
| miR398 | TGTGTTCTCAGGTCGCCCCCG | － | － | － |
| miR440 | AGTGTCTCCTGATGATCGGGACTA | － | － | － |
| miR530 | AGGTGCAGAGGCAGATGCAAC | － | － | － |
| miR535 | TGACAACGAGAGAGAGCACGC | － | － | － |
| miR806 | ACACGTGCTAAAAAGTCAACGGTG | － | － | － |
| miR812 | AAGACGGACGGTTAAACGTTGGAT | － | － | － |
| miR815 | GGAGGGGATTGAGGAGATTGGGAA | － | － | 0.79 |
| miR820 | TCGGCCTCGTGGATGGACCAGG | － | － | 0.66 |
| Ta-MIR501 | TTCGTGCCACGAACCGGTACTA | － | － | － |
| Ta-MIR502 | ACTACATTATGGAATGGAGGG | － | － | － |
| Ta-MIR504 | AACATTCTTATATTATGAGACGGA | － | － | 1.00 |
| Ta-MIR505 | AATATAAGAGCGTTTAGATCACTA | 2.02 | -0.94 | 0.00 |
| Ta-MIR506 | AATTTAGATACGGATGTATCTAGA | -0.30 | － | － |
| Ta-MIR507 | ATTCTATGAGACCAGGTCTCACGC | － | － | － |
| Ta-MIR509 | TTAACCAACGAGACCAACTGCGGC | － | -0.94 | 1.42 |
| Ta-MIR510 | CACTATGGACTACATACGGAGCAA | -0.31 | -0.53 | 1.01 |
| Ta-MIR511 | ACGACAAGTAATTCCGAACGGAAG | － | -2.27 | 1.00 |
| Ta-MIR512 | ATTTTCGGACGGAGGGAGTAGGGT | -0.30 | － | 1.02 |
| Ta-MIR514 | CTCCGTCTCGTAATGTAAGACG | － | － | － |
| Ta-MIR515 | TAAAGGTTAGCCACGAACCGGTAC | － | － | － |
| Ta-MIR516 | ATATTATGGAACGGAAGGA | － | － | － |
| Ta-MIR517 | TTTTCGGACGGAGGGAGTATA | － | － | － |
| Ta-MIR520 | AGATACATCCATACCTGCGACAAG | － | 0.47 | 1.01 |
| Ta-MIR521 | AAAGTTAGTACAAAGTTGAGTCAT | 1.42 | -0.07 | 0.95 |
| Ta-MIR523 | AGGTAGTAACATACACTAGTAACA | － | － | － |
