# Supplementary material for: Diverse set of microRNAs are responsive to powdery mildew infection and heat stress in wheat (Triticum aestivum L.)
Source: BMC Plant Biol. 2010 Jun 24;10:123. doi: 10.1186/1471-2229-10-123 (PMC3095282; doi:10.1186/1471-2229-10-123)
Supplement: Additional file 3 — Fold changes of new miRNA in response to wheat powdery mildew infection and heat stress. [file 1471-2229-10-123-S3.DOC]

Additional file 3 Fold changes of new miRNA in response to wheat powdery mildew infection and heat stress

| ID | new miRNA |  | Fold change(log2 JD8-*Egt*/JD-CK) | Fold change(log2 JD8-*Pm30-Egt*/JD*-Pm30*-CK) | Fold change(log2 TAM107-Heat/TAM107-CK) |
| --- | --- | --- | --- | --- | --- |
| Ta0639294 | Ta-miR2001 | TTGGACGAGGATGTGCAACTG | -4.66** | -0.82 | 0.39 |
| Ta0924479 | Ta-miR2002 | TTAGATGAGAAGGCAGATCATA | 1.09** | 0.62** | 0.08** |
| Tb7718 | Ta-miR2003 | TGACAAGTAATTTGGAACGGA | 1.02** | 0.98** | 0.32** |
| Ta0420822 | Ta-miR2004 | CATCTATTTTGGAACGGAGGG | 0.06** | 0.90** | 0.95 |
| Ta0881252 | Ta-miR2005 | TGAGAAGGCAGATCATAATAGC | 2.75** | -0.57 | 1.27** |
| Ta0533626 | Ta-miR2006 | TCTTATATTATGGGACGGAGG | -2.70** | -0.46 | -0.08** |
| Tb8128 | Ta-miR2007 | TTCTTATATTATGGGACGGAG | -1.43* | 1.21** | -0.04** |
| Tb7911 | Ta-miR2008 | AGACTTATATTTAGGAACGGA | -0.23 | 1.44** | 0.17** |
| Tb8291 | Ta-miR2009 | TGCAGTTGCTGTCTCAAGCTT | -0.21 | 0.25 | -0.21** |
| Tb8053 | Ta-miR2010 | AGACAAATATTTAGGAACGGA | 0.77** | 0.94** | 0.045** |
| Tb8182 | Ta-miR2011 | TTCCGAAAGGCTTGAAGCAAAT | -0.31 | 0.97** | -0.10** |
| Tb7888 | Ta-miR2012 | TCATCTATTTTGGAACGGAGG | 0.58 | 3.74** | 0.19** |
| Ta0636280 | Ta-miR2013 | TTGATGACAAGTATTTCCGGA | 4.16** | 0.58 | -0.32** |
| Ta0632144 | Ta-miR2014 | TTCTTATATTTTGGGACGGAG | -1.90 | 1.80* | -0.61** |
| Ta0632127 | Ta-miR2015 | TTCTTATATTGTGGGACGGAG | -1.00 | -0.14 | 0.69 |
| Ta0415768 | Ta-miR2016 | CAGAACCAGAATGAGTAGCTC | 0.28 | -0.15 | -0.13 |
| Ta0546827 | Ta-miR2017 | TCCGTCCGGAAATACTTGTCA | -0.31 | -2.65 | 1.46 |
| Ta0870192 | Ta-miR2018 | TCTTACATTATGGGACGGAGGG | － | -1.94 | － |
| Ta0408274 | Ta-miR2019 | ATTTTGGGACGGAGGGAGTAC | － | -1.53 | 0.16 |
| Ta0331147 | Ta-miR2020 | ACACTTATTTTGGACGGAGGG | － | 2.62 | -1.00 |
| Ta0270821 | Ta-miR2021 | AGCATGAGGCGACAACTGCAT | -1.89 | 2.04 | 0.00 |
| Ta0559250 | Ta-miR2022 | TCTATTTTAGAACGGAGGGAG | -0.63 | － | -0.80 |
| Ta0255645 | Ta-miR2023 | AAATAGATGACTCAACTTTAT | -2.63 | － | -1.58** |
| Ta1543767 | Ta-miR2024 | ACAAGTAATTCCGAACGGAGGGAG | 2.69* | 1.06 | 0.78 |
| Ta0534919 | Ta-miR2025 | TATTTCCGGACGGAGGGAGTA | -1.90 | － | -0.58 |
| Ta0514677 | Ta-miR2026 | TACTCCCTCCGTTCGGAATTA | － | － | 1.00 |
| Ta0492358 | Ta-miR2027 | ACTCCCTCCGTCCGGAAATAC | -1.90 | － | － |
| Ta0410873 | Ta-miR2028 | CAAGAATTTTGGGACGGAGGT | -0.90 | － | － |
| Ta0343240 | Ta-miR2029 | ACTCCCTCCGTTCGGAATTAC | -0.31 | -1.36 | 0.69 |
| Ta1200151 | Ta-miR2030 | TGATGACAAGTATTTTCGGACGG | － | 1.62 | 0.42 |
| Ta1007128 | Ta-miR2031 | AGACAAATATTTAGGAACGGAGG | 0.29 | － | -0.58 |
| Ta0843440 | Ta-miR2032 | TATATTATGAGACGGAGGGAGT | － | － | － |
| Ta0501511 | Ta-miR2033 | TAAACTGAGAGAGAGGGAGTA | － | 0.04 | － |
| Ta3154239 | Ta-miR2034 | TTTGACCAAAGCTAATATGCGGAC | － | － | － |
| Ta3055273 | Ta-miR2035 | TGGGCACAACCCACCAGGGCGCGC | -0.30 | － | － |
| Ta2864579 | Ta-miR2036 | GTTCTTATATTATGGGACGGAGGG | － | － | － |
| Ta2426709 | Ta-miR2037 | CTCTTATATTATGGGACGGAGGGA | 1.29 | 0.38 | -1.32 |
| Ta2270268 | Ta-miR2038 | CATCTATTTTAGAACGGAGGGAGT | 1.70 | 1.04 | 2.01 |
| Ta1431344 | Ta-miR2039 | AAGCGACGAGTAATATGGAACGGA | 1.29 | -0.94 | － |
| Ta1271787 | Ta-miR2040 | AAAATAGATGACTCAACTTTATAC | － | － | -0.99 |
| Ta1196315 | Ta-miR2041 | TGACAAGTATTTTCGGACGGAGG | -0.30 | － | － |
| Ta1179499 | Ta-miR2042 | TATATTATGGGACGGAGGGAGTA | － | － | － |
| Ta1103405 | Ta-miR2043 | CTCTGTCCCATAATGTAAGACGT | -0.30 | － | － |
| Ta1061452 | Ta-miR2044 | ATTTTGATGACAAGTATTTTCGG | － | － | 1.59 |
| Ta1028041 | Ta-miR2045 | AGTATTTCCGGACGGAGGGAGTA | 1.29 | － | 0.59 |
| Ta0623953 | Ta-miR2046 | TTATATTAGTTTACAGAGGGA | -0.30 | 0.04 | -0.86** |
| Ta0572119 | Ta-miR2047 | TGACAAGTAATTCGGGACGGA | － | － | － |
| Ta0533528 | Ta-miR2048 | TATTAGTTTACAGAGGGAGTA | 1.70 | 0.42 | -0.38** |
| Ta0528503 | Ta-miR2049 | TATATTCTGGGACAGAGGGAG | － | － | － |
| Ta0447988 | Ta-miR2050 | TATTTTGGAACGGAGGGAGTA | 0.70 | － | － |
| Ta0442645 | Ta-miR2051 | TATATTAGTTTACAGAGGGAG | 3.87 | 0.06 | -0.45** |
| Ta0402781 | Ta-miR2052 | ATTAGTTTACAGAGGGAGTAT | -0.30 | － | 0.00 |
| Ta0325262 | Ta-miR2053 | AATGTAAGACGTTTTTTGACA | -0.30 | － | 0.42 |
| Ta0321931 | Ta-miR2054 | AATATGGAACGGAGGGAGCAA | 1.29 | － | － |
| Ta0195095 | Ta-miR2055 | ATTATGGAAAGGAGGGAGTA | -0.30 | － | － |
| Ta1405846 | Ta-miR2056 | AAGACAAGTAATTCAGAACGGATG | － | － | 1.59 |
| Ta1289435 | Ta-miR2057 | AAACGCTCTTATATTATGGGACGA | － | － | － |
| Ta1270816 | Ta-miR2058 | AAAATAAGTGACTCAATTTTGTAC | － | － | － |
| Ta1196311 | Ta-miR2059 | TGACAAGTATTTCCGGACGGAGG | － | － | -1.00 |
| Ta1187145 | Ta-miR2060 | TCCGTTCCAAAATAGATGACCCA | － | － | － |
| Ta0944788 | Ta-miR2061 | TTTCTGCGACAAGTAATTCCGA | － | － | － |
| Ta0785843 | Ta-miR2062 | GAGTCATCTATTTTGGAACGGA | － | － | － |
| Ta0720052 | Ta-miR2063 | ATAATATAAGAACGTTTTTGAC | － | 1.62 | 1.11 |
| Ta0711764 | Ta-miR2064 | AGGATTGTAAAACAAATGGGGC | － | － | － |
| Ta0624100 | Ta-miR2065 | TTATATTTAGGAATGGAGGGA | － | － | － |
| Ta0607636 | Ta-miR2066 | TGTACAAATAAGCACCGGTGC | － | 0.64 | 0.01 |
| Ta0415609 | Ta-miR2067 | CACTTATTTTGAGACGGAGGG | － | 0.04 | 1.00 |
| Ta0415595 | Ta-miR2068 | CACTTATTATGGATCGGAGGT | － | 0.04 | － |
| Ta0391810 | Ta-miR2069 | CTATTTTGGAACGGAGGGAGT | － | 0.04 | － |
| Ta0345873 | Ta-miR2070 | ACTTACTTTGGGACGGAGGGA | － | 0.04 | － |
| Ta0326026 | Ta-miR2071 | AATTAGTTGACACTCAAACGG | － | -0.68 | 0.23 |
| Ta0263217 | Ta-miR2072 | ATTATGGGACGGAGGGAGTAG | － | － | － |
| Ta0608469 | Ta-miR2073 | TGTAGATACTCCCTAAGGCTT | -1.94** | -0.53 | 0.56 |
| Ta0835863 | Ta-miR2074 | TAGCTCCACTAAATTTGGAGCT | -1.90 | 1.04 | 0.62 |
| Ta0577216 | Ta-miR2075 | TGAGATGAGATTACCCCATAC | -0.14* | 0.67* | 0.14 |
| Ta0929045 | Ta-miR2076 | TTCCGAAAGGCTTGAAGCAAAT | 0.41* | 0.97 | -0.10** |
| Ta0586401 | Ta-miR2077 | TGCAGTTGCTGTCTCAAGCTT | -0.21 | 0.25 | -0.22** |
| Ta0564300 | Ta-miR2078 | TCTTATATTGTGGGACAGAGG | － | － | -0.59** |
| Ta0881324 | Ta-miR2079 | TGAGAAGGTAGATCATAATAGC | － | － | 0.57** |
| Ta0554274 | Ta-miR2080 | TCGGAATTACTTGTCGCGGAA | -0.20 | 0.28 | 0.41 |
| Ta0562274 | Ta-miR2081 | TCTGCGACGAGTAATTTGGAA | 0.91 | -0.53 | -0.73 |
