# Supplementary material for: Diverse set of microRNAs are responsive to powdery mildew infection and heat stress in wheat (Triticum aestivum L.)
Source: BMC Plant Biol. 2010 Jun 24;10:123. doi: 10.1186/1471-2229-10-123 (PMC3095282; doi:10.1186/1471-2229-10-123)
Supplement: Additional file 5 — The putative hairpin structures of new identified miRNAs. [file 1471-2229-10-123-S5.RTF]

Additional file 5 The Putative Hairpin Structures of New Identified miRNAs.
the letters in red indicate the miRNA sequence and the letters in green indicate the miRNA* sequence 

1 Ta-miR2001

      10        20        30         40        50     
U     CC   C          A  A         A   -|   A  G   GACC 
 ACGAU  UAG UAGAGCUUGG CG GGAUGUGCA CUG CGGU CG UAG    \
 UGCUG  AUC AUCUCGAACC GU CCUACACGU GAC GCCA GC AUC    U
-     UC   C          C  A         C   G^   -  -   GACU 
      100        90        80        70          60     

2 Ta-miR2002

      10        20        30        40        50 
GCC|                G             C       AAU   AG 
   GGUCUUUAGAUGAGAAG CAGAUCAUAAUAG UGAGCGA   GUA  \
   UCAGAAGUCUACUCUUC GUCUAGUAUUGUU ACUCGUU   CAU  U
---^                -             A       GC-   AG 
        90        80         70        60          

3 Ta-miR2003

        10        20        30        40        
                C   U    A  U            ACUAA 
CUCCGUUCCAAAUUACU GUC UAGA AU GAUGUAUCUAGA     \
GAGGCAAGGUUUAAUGA CAG GUUU Ta CUACAUAGAUCU     A
                A   U    A  C            ACAUA 
    90        80        70        60        50  

4 Ta-miR2004

        10        20        30        40   
|          C              C           C  A 
UACUCCCUUCG UCCAAAAUAGAUGA CCAACUUUGUA Ta A
AUGAGGGAGGC AGGUUUUAUCUACU GGUUGAAACAU AU G
^          A              U           A  U 
     80        70        60        50

5 Ta-miR2005

     10        20        30        40        50 
GCC|                G             C       AAU   AG 
   GGUCUUUAGAUGAGAAG CAGAUCAUAAUAG UGAGCGA   GUA  \
   UCAGAAGUCUACUCUUC GUCUAGUAUUGUU ACUCGUU   CAU  U
---^                -             A       GC-   AG 
        90        80         70        60          

6 Ta-miR2006

         10        20        30        40       
CUGAAA   -|  ACG  A                 UAAG  C   U 
      AUC AUA   AC CCUUCCGUCCCAUAAUA    AG GUU \
      UAG UAU   UG GGGAGGCAGGGUAUUAU    UC CAG U
AAA---   A^  AUA  A                 CACA  A   U 
90           80        70        60        50   

7 Ta-miR2007

   G                                C 
CCU UGUCCCAUAAUAUAAGAACGUUUUUGGCACUA \
GGA GCAGGGUAUUAUAUUCUUGUAAAAACUGUGAU A
   G                                C 
    70        60        50        40  

8 Ta-miR2008
        10        20        30         40        50         60        70       
                   A     C   U        C-    G            -   AAAU  GUGAAUCUAUA 
CCUCCGUUCCUAAAUAUAG UCUUU UAG GAUUUCAA  AGGU ACUACAUACGGA GUA    GA           \
GGAGGCAAGGAUUUAUAUU AGAAA AUC CUAAAGUU  UCUA UGGUGUAUGCCU CAU    CU           A
                   C     A   U        Ta    A            A   AUAU  GUAUAAAAUCC 
   150       140       130       120       110       100        90        80   


9 Ta-miR2009

        10        20        30        40        50    
    A       A         U                 AAAAUC     G 
CAAGC UGAGGCG CAACUGCAU ACUUGCAAGGAAGUUAC      UAUGG U
GUUCG ACUCUGU GUUGACGUG UGAACGUUCUUUCAGUG      AUACU C
    A       C         U                 CUA---     A 
   100        90        80        70           60   


10 Ta-miR2010

        10        20        30        40        50        60 
         A              C  A         CAA   AAAUA    U      A 
CUCCGUUCC AAAUAUUUGUCUUU Ta AGAUUUCAA   GUG     UGUC AUAUAC U
GAGGCAAGG UUUAUAAACAGAAA AU UCUAAAGUU   UAC     AUAG UGUAUG C
         A              A  C         ---   CUG--    -      U 
110       100        90        80             70        


11 Ta-miR2011

       10        20        30        40      
   A            GA      CG  A        AGA  Ta 
GGUU GCUUCAAGCCUU  GGAAUA  GU GACUGCAC   GC  \
UUAA CGAAGUUCGGAA  CCUUAU  CA UUGACGUG   CG  U
   A            AG      CA  G        A--  UG 
        80        70        60          50    

12 Ta-miR2012


        10        20        30        40     
          C                    A  C   C   AG 
CCCUCCGUUC AAAAUAGAUGACUCAAUUUU Ta UAG UUU  \
GGGAGGCAAG UUUUAUCUACUGGGUUGAAA AU AUU AAA  U
          A                    C  A   A   CA 
        80        70        60        50     

13 Ta-miR2013

        10        20        30        40        50         60      
A                 A     G          A            GU-|         A UAA 
 GGUACUCCCUCCGUCCG AAAUA UUGUCAUCAA AUGGAUAAAAGG   UGUAUCUAGA C   A
 UCAUGAGGGAGGCAGGC UUUAU AACAGUAGUU UACUUGUUUUCC   ACAUAGAUCU G   A
-                 C     G          C            ACU^         - CGU 
130       120       110       100        90        80         70   


14 Ta-miR2014

       10        20        30        40    
|   N      A                       C   CUAG 
UACU CCCUCC UCCCAAAAUAUAAGAAUGUUUUU ACA    \
AUGA GGGAGG AGGGUUUUAUAUUCUUGCAAAAA UGU    C
^   -      C                       C   AAUA 
      80        70        60        50      
 
15 Ta-miR2015

         10        20          30         
-----                        UCA--|     C   U 
     CUUCCUCCGUUCCACAAUAUAAGA     UUUCAA CUA \
     GAGGGAGGCAGGGUGUUAUAUUCU     AAAGUU GAU G
UUGAA                        UGCAA^     C   U 
   80        70        60        50        40 

16 Ta-miR2016

        10        20        30        40        50    
A|              C  C                    AU   CC    CA 
 AACCAUAGGAGGCGC UC AGAACCAGAAUGAGUAGCUC  GCA  UACU  A
 UUGGUAUCCUUCGCG AG UUUUGGUCUUACUCGUCGAG  UGU  GUGA  C
-^              U  A                    --   AA    Ta 
    100        90        80        70          60   

17 Ta-miR2017

       10        20        30        40            
UAC          C                   A   A     --|   AU 
   UCCCUCCGUC GGAAAUACUUGUCAUCAAA UGA UAAAA  UGGG  \
   AGGGAGGCAG CUUUUGUGAACAGUAGUUU ACU AUUUU  ACCU  A
Ta-          U                   A   A     CC^   AU 
.         90        80        70        60     

18 Ta-miR2018

          10        20        30        40         
--|   AG                    A   A                G 
  AGGA  UACUCCCUCCGUCCCAUAAU UAA ACAUUUUUUGACAUUA \
  UUUU  AUGAGGGAGGCAGGGUAUUA AUU UGUAAAAAACUGUGAU U
CA^   GG                    C   C                G 
        90        80        70        60        50 

19 Ta-miR2019

        10        20          30        40  
A                         --|             A 
 UACUCCCUCCGUCCCAAAAUAAGUG  UCAACUUUGUACUA C
 AUGAGGGAGGCAGGGUUUUAUUCAC  AGUUGAAACAUGAU U
C                         UG^             U 
     80        70        60        50      

20 Ta-miR2020

      10        20        30         40          
CUA|           U                    -    U      AG 
   CUCCCUCCGUCC AAAAUAAGUGUCUCAACUUU GUAC AACUUU  \
   GAGGGAGGCAGG UUUUAUUCACAGAGUUGAAA CAUG UUGAAA  U
---^           -                    U    -      CA 
       90         80        70        60    

21 Ta-miR2021

        10        20        30        40        50     
C|    A       A         U                 AAAAUC     G 
 CAAGC UGAGGCG CAACUGCAU ACUUGCAAGGAAGUUAC      UAUGG U
 GUUCG ACUCUGU GUUGACGUG UGAACGUUCUUUCAGUG      AUACU C
-^    A       C         U                 CUA---     A 
     100        90        80        70           60    

22 Ta-miR2022

       10        20        30        40        50       
AAA|   C              C  U         UC                 AG 
   CGAG UACUCCCUCCGUUC AA AUAGAUGAC  AACUUUAUAUUAACUUU  \
   GUUC AUGAGGGAGGCAAG UU UAUCUACUG  UUGAAAUAUGAUUGAAA  U
Ta-^   -              A  U         GA                 CA 
 .         100        90        80        70        60   

23 Ta-miR2023

        10        20        30        40      
UCA|     A                        C   A     A 
   UACUCC UCCGUUUCAAAAUAGAUGACUCAA UUU UACUA C
   AUGAGG AGGCAAGGUUUUAUCUACUGGGUU AAA AUGAU U
---^     G                        A   C     U 
           80        70        60        50   

24 Ta-miR2024

       10        20        30        40         50   
U   A     A    A            A       A    A    -|  GGU 
 ACU CUUUC UUCG AGUUACUUGUCG AAAAAUG AUGU UCUA GAU   U
 UGA GGAGG AAGC UUAAUGAACAGU UUUUUAC UGCA AGAU UUG   U
-   G     C    C            G       G    C    G^  AUU 
     100        90        80        70        60      

25 Ta-miR2025

        10        20        30        40        50        60        70      
|  U  UAU      G          Ta              A        C             C  AA  UAA 
GCU AU   GCAUAC CCCUCCGUCC  AAAUACUUGUCAUC AAAUGGAU AAAAGAGAUGUAU Ta  AC   A
CGA Ta   UGUAUG GGGAGGCAGG  UUUAUGAACAGUAG UUUACCUA UUUUCUCUACAUA AU  UG   A
^  U  UUU      A          CC              C        -             A  C-  CAU 
.       140       130       120       110       100         90         80   


26 Ta-miR2026

       10        20        30        40        50        60       
AAAU|AG  A       C                C     U  A                 ACUAA 
    C  CA UUCUACU CCUCCGUUCGGAAUUA UUGUC CG AAAUGGAUGUAUCUAGA     \
    G  GU AGGAUGA GGAGGCAAGCCUUAAU AACAG GU UUUACCUACGUAGAUCU     A
G---^GA  G       A                A     C  C                 ACAUA 
.          120       110       100        90        80        70   


27 Ta-miR2027

       10        20        30        40        50         60        70  
CUAAGAUA|              A             C        CA      -            ACUAA 
        UACUCCCUCCGUCCG AAAUACUUGUCAU AAAAUGGA  AAAAGG GAUGUAUCUAGA     \
        AUGAGGGAGGCAGGC UUUAUGAACGGUA UUUUACUU  UUUUCU CUACAUAGAUCU     A
UCACC---^              C             C        A-      A            ACAUA 
.          130       120       110       100         90        80        

28 Ta-miR2028

       10        20        30        40  
|   A    C    G                  UAG   UG 
AUAG UAUU CCUC GUCCCAAAAUUCUUGUCU   AUU  \
UAUU AUGA GGAG CAGGGUUUUAAGAACAGG   UAG  C
^   G    U    G                  CA-   AC 
 80        70        60        50         


29 Ta-miR2029

        10        20        30        40        50    
|            C       C        A                 ACUAA 
UACUCCCUCCGUU GGAAUUA UUGUCGCG AAAUGGAUGUAUCUAGA     A
AUGAGGGAGGCAA CCUUAAU AACAGCGU UUUACCUACAUAGAUUU     A
^            A       A        C                 GCCAU 
      100        90        80        70        60

30 Ta-miR2030

          10         20        30        40        50         60   
G--               -                            GA           -| AUA 
   UACUCCCUCCGUCCG GAAUACUUGUCAUCAAAAUGGAUAAAAG  GAUGUAUCUAG AC   U
   AUGAGGGAGGCAGGC UUUAUGAACAGUAGUUUUAUCUAUUUUU  UUACAUAGAUC UG   U
AUG               U                            AC           U^ AUU 
.       120       110       100        90        80        70     


31 Ta-miR2031

        10        20        30        40        50        60       
|              A              C  A         CAA   AAAUA    U      A 
UACUCCCUCCGUUCC AAAUAUUUGUCUUU Ta AGAUUUCAA   GUG     UGUC AUAUAC U
AUGAGGGAGGCAAGG UUUAUAAACAGAAA AU UCUAAAGUU   UAC     AUAG UGUAUG C
^              A              A  C         ---   CUG--    -      U 
      120       110       100        90           80           70  


32 Ta-miR2032

        10        20        30         40         50 
CAU       NUA                CG      -   G-|    UUUU 
   AUUCCCU   GUCUCAUAAUGUAAGA  UUUUUU ACA  ACGUU    A
   UGAGGGA   CAGAGUAUUAUAUUUU  GAAAAA UGU  UGUAG    A
---       GG-                AU      C   GA^    UCAC 
 100         90        80        70        60        

33 Ta-miR2033

       10        20        30        40        50        60        70        80        90       100         110  
UAC         CG          C   UCC        Ta                U    C          AC  U   GC       U       UU-    AA-| GAA 
   UACUCCCUC  UUUCAGUUUA AAG   UGUGCGUA  CCUAGGUUGUCAAUUU AUCA CCUAAUAUAA  Ta AUA  ACAAAAA UAUACCG   UGAA   Ta   \
   AUGAGGGAG  AGAGUCAAAU UUC   ACGCGCAU  GGAUCCAGCAGUUAAA UGGU GGAUUAUGUU  GU UAU  UGUUUUU AUAUGGU   AUUU   GU   C
---         AG          A   CGC        GG                C    U          CA  U   AA       U       UAU    GAA^ AUA 
   220       210       200       190       180       170       160       150       140       130       120        

34 Ta-miR2034

      10        20        30        40        50        60        70        80        90       100       110       120        
|            C              G    AC         A     C   G      C  A        A   U    A     U   C    C     U    G  GUGAAAG  C     A 
CUCCGUUUUUAUU AGUCUGCAUAUUAG UUUG  CGAAGUCAA CUUUG AAA UUUGAC AA UUUAUAGA AAA AUAU AACAU UAC AUAA AAAUC AUAU AU       Ta AUUCA U
GAGGCAAAAAUAA UCAGGCGUAUAAUC AAAC  GUUUCAGUU GAAAC UUU AAACUG UU AAAUAUUU UUU UAUA UUGUA AUG UAUU UUUAG UAUG UG       AU UAAGU A
^            A              G    CA         C     A   G      A  C        G   U    A     U   U    U     U    G  -------  C     A 
       240       230       220       210       200       190       180       170       160       150       140              130 

35 Ta-miR2035

    10        20        30        40        
-|      G  AC      U     C                  UGAG 
 CUGGGGG GC  CCGAGG GGGCA AACCCACCAGGGCGCGCC    C
 GACCCCC CG  GGCUCC CUCGU UUGGGUGGUCCCGCGCGG    C
G^      A  A-      U     A                  UCCU 
     90         80        70        60        50 

36 Ta-miR2036

        10        20        30        40       
AACA|                          UG     U      C 
    UACUCCCUCCGUCCCAUAAUAUAAGAG  UUUUU ACACUA \
    AUGAGGGAGGCAGGGUAUUAUAUUCUU  AAAAA UGUGAU A
UG--^                          GG     C      U 
90          80        70        60        50   


37 Ta-miR2037

      10        20        30         
|                C           UU      G 
CCCUCUGUCCUAUAAUA AAGAGCGUUUU  ACACUA \
GGGAGGCAGGGUAUUAU UUCUCGCAAAA  UGUGAU U
^                A           CC      G 
      70        60        50        40 

38 Ta-miR2038

        10        20        30        40     
AACUA|                                     C 
     UCUCUCCGUUCUAAAAUAGAUGAUUUAAUUUUGUACUA A
     AGGGAGGCAAGAUUUUAUCUACUGAGUUGAAACAUGAU G
UG---^                                     U 
         80        70        60        50    


39 Ta-miR2039

        10         20        30        40        50        60    
UCUUCUCU     -| CGUACA                              G          A 
        GCUGC CU      UACUCCCUCCGUUCCAUAUUACUCGUCGCU AUUUGGUACA A
        CGACG GA      AUGAGGGAGGCAAGGUAUAAUGAGCAGCGA UAAAUCAUGU G
UCUUUCUU     U^ ACUCAG                              A          U 
       120       110       100        90        80        70     

40 Ta-miR2040
         10        20        30        40        
-|                                Ta          AG 
 ACUCCCUCUGUUCCAAAAUAGAUGACUCAACUU  UACUAACUUU  \
 UGAGGGAGGCAAGGUUUUAUCUACUGGGUUGAA  AUGAUUGAAA  U
A^                                GC          CA 
     90        80        70        60        50  


41 Ta-miR2041

        10        20        30        40        50        60        70  
CAACUUAC|                    G                               C    UGUAU 
        UACUCCCUCCGUCCGGAAAUA UUGUCAUCAAAAUGGAUAAAAGAGGAUGUAU UAGA     \
        AUGAGGGAGGCAGGCUUUUAU AACAGUAGUUUUACCUAUUUUCUUCUACAUA AUCU     U
C-------^                    G                               A    UGAUU 
           130       120       110       100        90        80        

42 Ta-miR2042

         10        20        30        40      
---| C                 U               U       C 
   AG UACUCCCUCUGUCUCAU AUGUAAGAUGUUUUU GACGCUA \
   UC AUGAGGGAGGCAGGGUA UAUAUUCUGCAAAAA UUGUGAU A
UAA^ -                 U               -       C 
 90         80        70        60         50    

43 Ta-miR2043

         10        20        30          
CUA-|                A               CAA 
    CUCUGUCCCAUAAUGUA GACGUUUUUGCAGUU   \
    GAGGCAGGGUAUUAUAU CUGCAAAAACGUCAA   U
GAGG^                C               AUU 
.        70        60        50          

44 Ta-miR2044

       10        20        30        40        50        60         
A|                                    C              U    C    UGUAU 
 UAUAUACUUCCUCCGUCCGGAAAUACUUGUCAUCAAA UGGAUAAAAGGAGA GUAU UAGA     \
 AUAUAUGAGGGAGGCAGGCUUUUAUGAACAGUAGUUU ACCUAUUUUUCUCU CAUA AUCU     U
A^                                    U              C    A    UGAUU 
     130       120       110       100        90        80        70 

45 Ta-miR2045

        10        20        30        40        50        60     
|               A   A                       A  C      C    ACUAA 
AUACUCCCUCUGUCUG AAA ACUUGUCAUCAAAAUGAAUAAAA GG AUGUAU UAGG     \
UAUGAGGGAGGCAGGC UUU UGAACAGUAGUUUUACUUAUUUU CC UACAUA AUCU     U
^               C   A                       C  C      A    ACAUA 
       120       110       100        90        80        70     

46 Ta-miR2046

       10        20        30        40        
ACAUG|        CA                         U    C 
     UACUCCCUC  UAAAUUAAUAUAAGAGCGUUUAGAU ACUA \
     AUGAGGGAG  AUUUGAUUAUAUUCUUGCAAAUCUA UGAU U
CAUA-^        AC                         U    U 
    90        80        70        60        50  

47 Ta-miR2047

  10        20        30        40        50        60  
    AU-|  A         C       C         GC            C    AUUAA 
GUGA   GGU CUCCCUCCG CCCGAAU ACUUGUCAU  AAAUGGAUGUAU UAGA     A
CAUU   CCA GAGGGAGGC GGGCUUA UGAACAGUG  UUUACUUACAUA AUUU     U
    CAU^  C         A       A         Ta            A    ACAUA 
   120       110       100        90        80        70       

48 Ta-miR2048

       10        20        30        40  
CAAC|         A                         C 
    UACUCCCUUU UAAAUUAAUAUAGGAUCGUUUAGAU C
    AUGAGGGAGA AUUUGAUUAUAUUCUAGCAAAUUUA C
U---^         C                         U 
   80        70        60        50       


49 Ta-miR2049

      10        20        30        40        50  
GAU| G  G         C      A                 A   CUAG 
   AU AA UACUCCCUC GUCCCA AAUAUAAGAACGUUUUU ACA    \
   UG UU AUGAGGGAG CAGGGU UUAUAUUCUUGCAAAAA UGU    C
---^ G  G         A      C                 C   CAUA 
 100        90        80        70        60   


50 Ta-miR2050

       10        20        30        40      
UCA|     A                        C   A     A 
   UACUCC UCCGUUUCAAAAUAGAUGACUCAA UUU UACUA C
   AUGAGG AGGCAAGGUUUUAUCUACUGGGUU AAA AUGAU U
---^     G                        A   C     U 
           80        70        60        50   


51 Ta-miR2051

      10        20        30        40    
CU|       C                             CUA 
  ACUCCCUC GUAAACUAAUAUAAGAGUGUUUAGAUUAC   \
  UGAGGGAG CAUUUGAUUAUAUUCUCACAAAUCUAGUG   U
--^       A                             AUU 
      80        70        60        50      

52 Ta-miR2052

       10        20        30        40        50    
AUGAU|   U          C            C            A     A 
     ACUA AUGCUCCCUC GUAAACUAAUAU AGAGCAUUUAGA UACUA \
     UGAU UAUGAGGGAG CAUUUGAUUAUA UCUCGUAAAUCU GUGAU A
ACAUU^   -          A            U            A     G 
    100         90        80        70        60      

53 Ta-miR2053

         10        20         30         40 
CA-  CU          U      -             -| AA 
   UC  UCUCAAAAUG AAGACG UUUUUUGACACUA CA  U
   AG  AGAGUUUUAC UUCUGU AAAAAACUGUGAU GU  C
GGA  AC          C      A             A^ AU 
    80        70        60        50        


54 Ta-miR2054

        10        20        30        40  
|   A                  AAC     C        U 
AAUU CUCCCUCCGUUCCAUAUU   UGUCG UGAUUUAG G
UUAA GAGGGAGGCAAGGUAUAA   ACAGC ACUAAAUC U
^   C                  AUC     A        A 
   80        70        60        50      


55 Ta-miR2055

           10        20        30        40        50  
-    AA--   -|         GUC              Ta            C 
 GGUG    AAA UACUCCCUUU   CCAUAAUAUAAGAA  UUUUUGACACUA \
 CCAU    UUU AUGAGGGAGG   GGUAUUAUAUUCUU  AAAAACUGUGAU A
C    AAAG   C^         AAA              GC            C 
     100        90        80        70        60        

56 Ta-miR2056

       10        20        30        40        50          
  C       C                    A         AUCC       A-|  CA 
CC UCCGUUC GAAUUACUUGUCUUGGAUUU UCUAGAUAC    GUAUCUA  AUU  U
GG AGGCAAG CUUAAUGAACAGAAUCUAAA AGAUCUAUG    CAUAGAU  UGA  U
  U       A                    C         CCUA       CG^  UU 
       110       100        90        80        70        60


57 Ta-miR2057

        10        20        30      
CUC                             CUC 
   CGUUCCAUAAUAUAAGAGCGUUUUUGACA   A
   GCAGGGUAUUAUAUUCUCGCAAAAACUGU   U
AA-^                            AAU 
70         60        50        40   


58 Ta-miR2058

         10        20        30        40        50    
UC-        C          A         C    U          C   AG 
   AGAUACUC UUCUGUCUCA AAUAAGUGA UCAA UUUGUACUAA UUU  \
   UUUAUGAG GAGGCAGGGU UUAUUCACU AGUU AAACAUGAUU AAA  U
UUU^       A          C         A    C          A   CA 
      100        90        80        70        60   

59 Ta-miR2059

        10        20        30        40        50        60       
UGU               A                      C    AU        A    ACUAA 
   UACUCCCUCCGUCCG AAAUACUUGUCAUCAAAAUGGA AAAA  GGAUGUAU UAGA     \
   AUGAGGGAGGCAGGC UUUAUGAACAGUAGUUUUACUU UUUU  CCUACAUA AUCU     A
UGC^              C                      A    CC        G    ACAUA 
 130       120       110       100        90        80        70   


60 Ta-miR2060


        10        20        30        40          
AUUUU|                            C   C   A   UAA 
     CGUACUCCCUCCGUUCCAAAAUAGAUGAC CAA UUU UAC   \
     GUAUGAGGGAGGCAAGGUUUUAUCUACUG GUU AAA AUG   C
UUCAU^                            A   A   C   UUU 
        90        80        70        60   

61 Ta-miR2061

        10        20        30        40        50  
           C              U  A               AA   AA 
 CUCCCUCCGU CGGAGUUACUUGUC CG AAAUGGAUGUAUUUA  ACU  A
 GAGGGAGGCA GCCUUAAUGAACAG GU UUUACCUAUAUAGAU  UGG  A
U^         A              C  C               C-   AU 
  100        90        80        70        60    


62 Ta-miR2062

        10        20        30        40        50 
                             G       A  C       AG 
GCUACUCCCUCCGUUCCAAAAUAGAUGAC CAACUUU Ta UAACUUU  \
UGGUGAGGGAGGCAAGGUUUUAUCUACUG GUUGAAA AU AUUGAAA  U
                             A       C  A       CA 
 .        90        80        70        60   


63 Ta-miR2063

        10        20        30        40 
-|   C                A                 U 
 CUCC UCCGUCCCAUAAUAUA GAACGUUUUUGACACUA \
 GAGG AGGCAGGGUAUUAUAU CUUGCGAAAAUUGUGAU A
U^   U                C                 C 
 80        70        60        50   


64 Ta-miR2064

         10        20        30        40        50        60    
     -|  UAGC             A    C    U                 C   A    A 
CCCCU CUC    UUCUCCUAGAAUC UUGA CCCC UUUGUUUUACAAUCCUA CUA UUAG \
GGGGA GGG    AAGAGGGUUUUAG AACU GGGG AAACAAAAUGUUAGGAU GAU AAUC C
     U^  CCU-             A    C    U                 A   C    C 
     120        110       100        90        80        70 


65 Ta-miR2065

           10        20        30        40        50        
          C         C           C     C   A  A        A  GAG 
   CCCUCCA UCUUAAAUA AAGUCUUUCUA AGAUU CAA AG GACUACAU CG   G
   GGGAGGU AGGAUUUAU UUCAGAAAGAU UCUAA GUU UC CUGAUGUA GU   A
UGA       A         A           C     A   A  C        A  AAA 
       110       100        90        80        70        60 


66 Ta-miR2066

          10        20        30         
--|    G          UUC          U      CG 
  UUCCU AAGCACCGGU   UAUUUGUACA GAUAGA  A
  AAGGA UUCGUGGCCA   AUAAACAUGU UUAUCU  G
GA^    G          CGA          C      AU 
.        70        60        50        40


67 Ta-miR2067

        10         20        30        40        
GA|  A         A-  C                           A 
  AUG UACUCCCUC  UC CAAAAUAAGUGUCGUUGAUUUAGUACA C
  UAC AUGAGGGAG  AG GUUUUAUUCACAGUGAUUAAAUCAUGU U
A-^  -         GC  A                           U 
      90        80        70        60        50 


68 Ta-miR2068

          10         20        30        40  
--          -|      U  U                  AG 
  CUCCGAUCCA AAUAAGU UC CAGUUUUGAACUAAGGUU  \
  GAGGCUAGGU UUAUUCA AG GUCAAAACUUGGUUCCAA  U
UG          A^      C  C                  CU 
     80        70        60        50       


69 Ta-miR2069

       10        20        30        40   
|          C              C           C  A 
UACUCCCUUCG UCCAAAAUAGAUGA CCAACUUUGUA Ta A
AUGAGGGAGGC AGGUUUUAUCUACU GGUUGAAACAU AU G
^          A              U           A  U 
     80        70        60        50

70 Ta-miR2070

        10        20        30        40          
AC|      A       A                          UGUAC 
  UCCCUCC UCCCAAA UAAGUGUCUCAACUUUAGUACAAAGU     \
  AGGGAGG AGGGUUU AUUCACAGAGUUGAAAUCAUGUUUCA     U
--^      C       C                          UGAAA 
        90        80        70        60          


71 Ta-miR2071


       10        20        30        40        50     
-|    UCC               U    A                   C  UGA 
 AUACU   UCCGUCUGGAAUUAG UGAC CUCAAACGGAUGUAUCUAG AC   A
 UAUGA   AGGUAGGCCUUAAUC ACUG GAGUUUGCCUACAUAGAUC UG   A
A^    UGA               U    C                   -  CAU 
.       100        90        80        70         60  


72 Ta-miR2072

        10        20        30        40     
U|                         U           U   A 
 UACUGCUUCCUUCGUCCCAUAAUAUA GAGUGUUUUUU UUU C
 AUGAUGAGGGAGGCAGGGUAUUAUAU CUCGCAAAAAA GAA A
C^                         U           U   A 
.        80        70        60        50    


73 Ta-miR2073
            10        20        30        40        50          
----|  A  UU  AG    AAU          U  A        CUAG     U    UUCA 
    CAA GC  UA  CACU   UAAGCCUUAG GA UAUCUACA    UAAGU UUUC    \
    GUU CG  AU  GUGG   GUUCGGAAUC CU AUAGAUGU    AUUCG AAAG    U
GUGU^  G  UU  G-    ---          C  C        UACG     U    UUUG 
       110           100        90        80        70          

74 Ta-miR2074
        10        20        30        40      
G|       C      C     A            CGCC    AC 
 UUCGAUUC UAGCUC ACUAA UUUGGAGCUUUA    AGCG  \
 AGGUUGAG AUCGAG UGAUU AAACCUCGAAAU    UUGC  C
A^       A      -     C            UC--    Ta 
       80         70        60          50    

75 Ta-miR2075
         10         20        30        40        50       
UUU-   U   U-|     C      A       A    A   CCUAUA    C  CU 
    CUC CUG  AGCGGC GUUGAG UGAGAUU CCCC UAC      GCGG AG  \
    GAG GAC  UCGUCG CAACUC ACUCUAA GGGG AUG      CGCC UC  C
UUUC   U   UC^     A      C       C    G   CGCACA    -  AG 
  110       100        90        80        70         60   


76 Ta-miR2076
        10        20        30        40        50     
C|  AC       A            GA      CG  A        AGA  Ta 
 UGC  GUUGGUU GCUUCAAGCCUU  GGAAUA  GU GACUGCAC   GC  \
 ACG  CAAUUAA CGAAGUUCGGAA  CCUUAU  CA UUGACGUG   CG  U
U^  AA       A            AG      CA  G        A--  UG 
     100        90        80        70        60       


77 Ta-miR2077
        10        20        30        40        50        60   
U| UGG  C     A       A         U      A          AAAAUC     G 
 GG   CA CAAGC UGAGGCG CAACUGCAU ACUUGC AGGAAGUUAC      UAUGG U
 CC   GU GUUCG ACUCUGU GUUGACGUG UGAACG UCUUUCAGUG      AUACU C
G^ UCA  C     A       C         U      C          CUA---     A 
 120       110       100        90        80           70      


78 Ta-miR2078
        10        20        30        40        50        60  
UCUUU|        C                  A    CGUCC   A    AA   C   C 
     UACUCCCUC GUCCCAUAAUAUAAGAGC UUUU     CAU AUAU  GAG GUU \
     AUGAGGGAG CAGGGUGUUAUAUUCUCG AAAA     GUG UGUG  UUC CAA C
UUUAC^        A                  G    ACU--   A    A-   A   U 
.       110       100        90        80           70       


79 Ta-miR2079
        10        20         30        40          50 
UUG       A             -                 .-A      Ta 
   UUGCUCA UUGUUAUGAUCUG CUUCUCAUUUGAAGACU   GUUUAU  \
   AGCGAGU GAUAAUACUAGAU GAAGAGUAGAUUUCUGA   CGAAUA  C
UAA       C             G                  -      Ta 
 210       200       190       180                    

80 Ta-miR2080
         10        20        30        40        50  
-|                        G        A           UGUAU 
 UUCUCUCCGUUCGGAAUUACUUGUC CGGAAAUG AUGUAUCUAGA     \
 GAGGGAGGCAAGCCUUAAUGAACAG GCCUUUAC UACAUAGAUUU     U
U^                        A        C           UGACU 
  100        90        80        70        60  
 
81 Ta-miR2081
        10        20        30        40        50    
U|       CG                   UGUAUG            UGUAU 
 ACUCCCUC  UUCCGAAUUACUUGUCGCA      GAUGUAUCUAGA     \
 UGAGGGAG  AAGGUUUAAUGAGCAGCGU      CUACAUAGAUCU     U
-^       AA                   CUUUAA            UGAUU 
     100        90        80        70        60      
    


  


   


 


      


  
