# Supplementary material for: Diverse set of microRNAs are responsive to powdery mildew infection and heat stress in wheat (Triticum aestivum L.)
Source: BMC Plant Biol. 2010 Jun 24;10:123. doi: 10.1186/1471-2229-10-123 (PMC3095282; doi:10.1186/1471-2229-10-123)
Supplement: Additional file 6 — New identified miRNAs by 4× depth Brachypodium distachyon genomic sequences. [file 1471-2229-10-123-S6.DOC]

Additional file 6 New identified miRNAs by 4x depth *Brachypodium distachyon* genomic sequences

| Name | Sequence | Length(nt) | Free Energy  (kcal/mol) | Genome Location |
| --- | --- | --- | --- | --- |
| brachy-miR1 | GCGTGCAAGGAGCCAAGCATG | 21 | -57.4 | super_0:10869211:10869334 |
| brachy-miR2 | ACAATTATTTAGGAACGGAGG | 21 | -38.6 | super_0:20867531:20867629 |
| brachy-miR3 | ACTTATTTTGGGATGGAGGGA | 21 | -56.8 | super_13:507557:507656 |
| brachy-miR4 | TCATTTATTTTGGAACGGAGGGA | 23 | -52.9 | super_15:1368371:1368485 |
| brachy-miR5 | ATTAATTTGGATCGGAGGGA | 20 | -88.1 | super_1:28350393:28350536 |
| brachy-miR6 | TTGAACTAAAGAGGGTCGGAG | 21 | -57.2 | super_2:2472878:2472995 |
| brachy-miR7 | ACAGTTATTTTGGGACGGAGG | 21 | -47.3 | super_4:23764691:23764801 |
| brachy-miR8 | TGTAGATACTCCCTAAGGCTT | 21 | -48.1 | super_505:681:781 |
| brachy-miR9 | TCAATTAATTTGGATCGGAGGGAGT | 24 | -85.8 | super_5:2057465:2057605 |
| brachy-miR10 | AATTAATTTGGATCGGAGGGA | 21 | -54.6 | super_6:14911255:14911397 |
| brachy-miR11 | AGACAACTATTTAGGAACGGA | 21 | -36.1 | super_6:14274058:14274132 |
| brachy-miR12 | ACTTATTTTGGGATGGAGGGA | 21 | -56.8 | super_13:507557:507656 |
| brachy-miR13 | TAAATATTTAGGAACGGAGGG | 21 | -45.6 | super_13:1281620:1281699 |
| brachy-miR14 | CATTTATTTTGGAACGGAGGG | 21 | -52.9 | super_15:1368371:1368485 |
| brachy-miR15 | AACAACTAATATGGAACGGAG | 21 | -59.3 | super_1:11291028:11291142 |
| brachy-miR16 | AAATATTTAGGAACGGAGGGA | 21 | -45.2 | super_1:30448360:30448461 |
| brachy-miR17(Ta-miR2019) | ATTTTGGGACGGAGGGAGTAC | 21 | -64.9 | super_14:2242276:2242384 |
| brachy-miR18(Ta-miR2050) | TATTTTGGAACGGAGGGAGTA | 21 | -50.1 | super_13:4095655:4095722 |
| brachy-miR19(Ta-miR2070) | ACTTACTTTGGGACGGAGGGA | 21 | -64.8 | super_14:307685:307587 |
| brachy-miR20(Ta-miR2072) | ATTATGGGACGGAGGGAGTAG | 21 | -54.9 | super_200:3297:3356 |
