# Supplementary figures and images for: Diverse set of microRNAs are responsive to powdery mildew infection and heat stress in wheat (Triticum aestivum L.)
Source: BMC Plant Biol. 2010 Jun 24;10:123. doi: 10.1186/1471-2229-10-123 (PMC3095282; doi:10.1186/1471-2229-10-123)

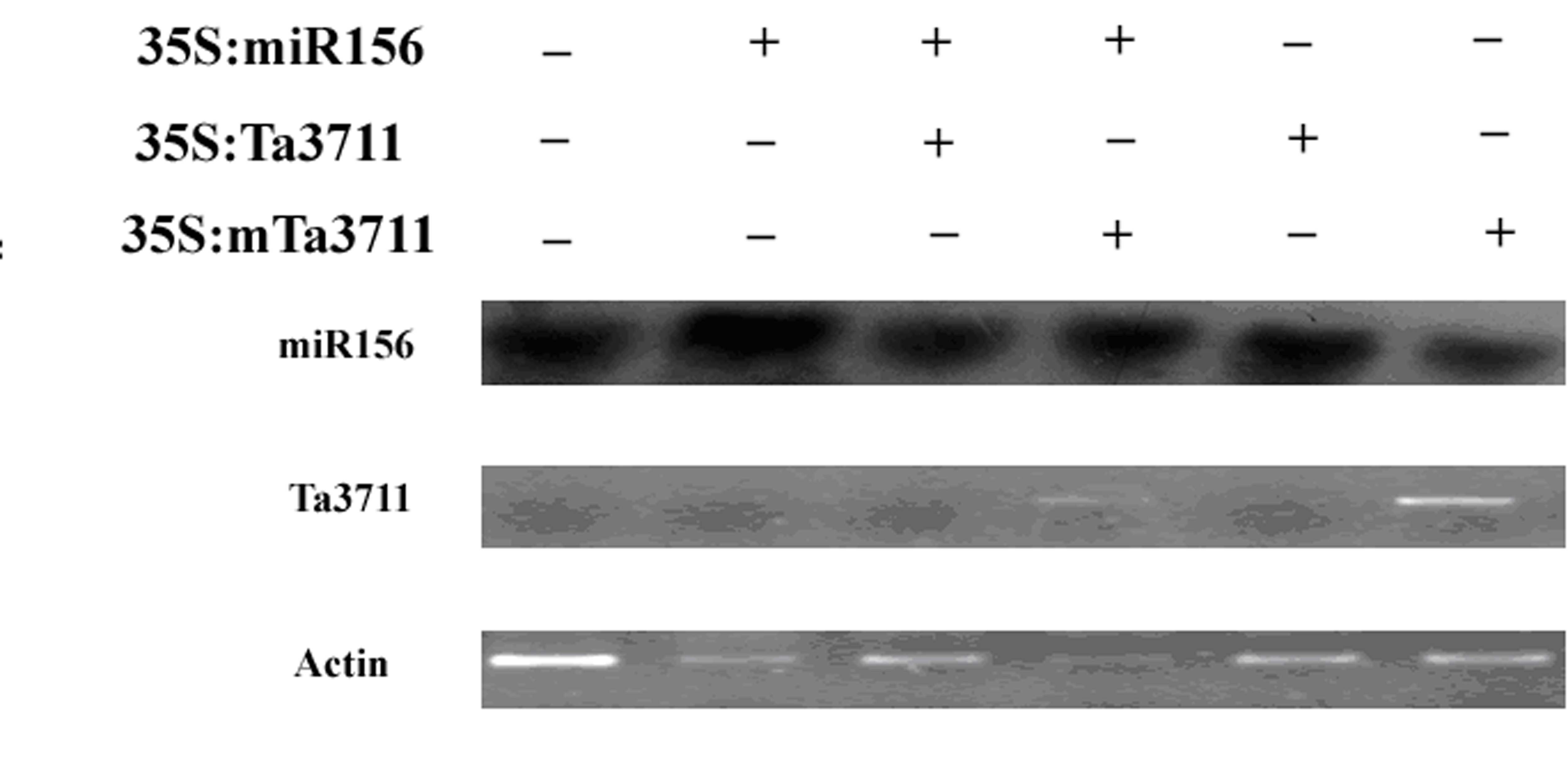

Supplement: Additional file 8 — Figure S1 MiR156 directs the cleavage of Ta3711 transcripts. [file 1471-2229-10-123-S8.JPEG]
