# Supplementary material for: Diverse set of microRNAs are responsive to powdery mildew infection and heat stress in wheat (Triticum aestivum L.)
Source: BMC Plant Biol. 2010 Jun 24;10:123. doi: 10.1186/1471-2229-10-123 (PMC3095282; doi:10.1186/1471-2229-10-123)
Supplement: Additional file 9 — Primer sequences of miRNA target genes used for RT-PCR analysis. [file 1471-2229-10-123-S9.DOC]

| Name of Target Genes | Primer sequences |
| --- | --- |
| Ta3711-left | ACCCAGGAGGCTTCAAAGTT |
| Ta3711-right | CAGAGTGCATCTGGGGACTT |
| Ta7012-left | CTCATCTACCCAATGCTGTTC |
| Ta7012-right | CGGTTTACGCCAAAGAAAGT |
| Ta38051-left | GCACCGTGTTTCTGAGAG |
| Ta38051-right | CCATTCGAGCCCACTTATTC |
| Ta6394-left | GAGTTACGTGGTGAGCTGGA |
| Ta6394-right | TCCGTTTCAGTAGTCCAGTGG |
| Ta93981.-left | GCCATGTTCACACACTCCTG |
| Ta9398.1-right | GCTTTCCAGTTCCGGGGTC |
| Ta9398.2-left | GGCACGAGAATCAACCACCT |
| Ta9398.2-right | CTCTTCAGAACTCGACCTCGA |
| Ta9550-left | TGTACGGCATTAGGGGTCAG |
| Ta9550-right | CCGGACGGAGATAGGTAACG |
| Ta29451-left | TGATGTCGGCTTTGTCTCTG |
| Ta29451-right | AGGTACGGCTTACGGCTTTT |
| Ta8589-left | ATCATCACGATTCCCACCCTA |
| Ta8589-right | CACATCCATCCGTCCTCTCA |
| Ta24445-left | CCACTCGCCGTTGCTTTAC |
| Ta24445-right | GTCACCAAAACATGGCTTGG |
| Ta13336-left | GCCACTCTTCTCGTCGTCAT |
| Ta13336-right | GCAAGACCGAAAACGCTGAA |

Additional file 8 Primer sequences of miRNA target genes used for RT-PCR
